# An interactive information based DCNN-BiLSTM model with dual attention mechanism for facial expression recognition

**DOI:** 10.1038/s41598-025-09709-1

**Published:** 2025-07-19

**Authors:** Samanthisvaran Jayaraman, Anand Mahendran

**Affiliations:** 1 School of Computer Science and Engineering, Vellore Institute of Technology, Vellore, Tamilnadu India; 2 School of Computer Science and Engineering, Vellore Institute of Technology, Chennai campus, Vellore, Tamilnadu India

**Keywords:** Facial expression recognition, Histogram of oriented gradients, DCNN-BiLSTM, Dual attention mechanism with cross-fusion, Interactive learning information, Engineering, Mathematics and computing

## Abstract

Human’s facial expressions and emotions have direct impact on their action and decision-making abilities. Basic CNN models are complexity of speeding up the operation to minimize the complexity. In this paper, we have proposed a Deep Convolutional Neural Networks along with Bi-Long Short Term Memory, which is followed by a single and cross-fusion attention mechanism for gathering both spatial and channel information from feature vector maps. Piecewise Cubic Polynomial and linear activation function was used to speed up Interactive Learning Information (ILI). Global Average Pooling (GAP) computes weights for feature vector maps; softmax classifier is used to classify images into 7 classes based on the expression present on the input images. The proposed model’s performance was compared with benchmarking methods like NGO-BiLSTM, ICNN-BiLSTM and HCNN-LSTM. The proposed model resulted with better accuracy than other methods with 82.89%, 96.78%, 95.78%, and 95.87% on FER 2013, CK+, RAF-DB and JAFFE datasets and also resulted in lower False Recognition Rate (FAR) of 7.23%, 1.42%, 1.96% and 1.78% on all four datasets respectively. The proposed model has performed well than other benchmarking models with high Genuine Recognition Rate (GAR) of 88.57% on FER2013, 97.23% on CK+, 96.87% on RAF-DB and 96.32% on JAFFE datasets respectively.

## Introduction

With ever evolving advancements in Artificial Intelligence has helped the research experts to utilize intelligent information systems and deep learning models to excel in the field of health care, transportation systems, security and Facial Expression Recognition (FER) more. Advanced optimization techniques plays crucial role in various disease diagnosis such as liver cancer detection, kidney disease diagnosis and brain tumor, etc., Diagnostic tools like X-ray, Coherence Tomography (CT), Magnetic Resonance Imaging (MRI) offers valuable insights in disease diagnosis with advanced attention mechanisms for improved classification accuracy on diverse applications like Facial Emotions Classification of humans to ensure road safety or asses their state behavior, kidney disease classification and Acute Lymphoblastic Leukemia Detection to ensure patient’s health state and coffee bean quality classification in beverage industry and more^1, 2, 3^.

Initially, psychologists has started their research on facial expression of their clients which encouraged the researchers and scholars to pay attention to Facial Expression Recognition (FER) this topic with ever growing artificial intelligence and advancements in computer vision, automatic recognition for detecting facial expressions of an image or from videos^4^. Since Facial Expression Recognition (FER) has become an important research topic, Deep learning based approaches are most widely used by the researchers in the recent past for FER. Facial features and Face model features are the most widely used models for FER. Facial Features pays attention to features like eye, mouth and eyebrows and etc., whereas Face Model Features takes these facial features to model the face and recognize the expression of an image^5^.

Although, human being express their emotions in many ways such as language, voice and facial expression, facial expressions holds large amount of rich and effective information as it conveys what really goes through their hearts. At times it is more accurate than other expressions such as language and voice tone. In recent years, public have started using more AI devices in their daily life such as home, office and public places, the hope among public is increasing that computers could understand their emotions and expression better^6, 7^.

Facial expression recognition as a classification problem, most tradition methods widely uses hand-crafted features like Local Binary Patterns (LBP), Gabor Transform and traditional ML based methods like Support Vector Machines (SVM) used for classification. With uncontrollable environmental or wild data sets like FER 2013 and FER plus, achieving high accuracy is difficult for such methods with spatial and temporal information. However, when deep learning based approaches were adopted for FER classification, the effectiveness of results and accuracy has improved significantly^4, 8^.

Deep learning methods like Convolutional Neural Networks (CNN) has been widely adopted in many studies which mainly uses universal non-linear fitting tools for input image data and use Back Propagation (BP) to learn posterior probability function to address classification problem. Moreover, CNN uses manually designed architectures such as Alexnet, VGGNet and ResNet and DenseNet and to obtain more discriminative deep features softmax loss function and center loss functions have been used to supervise CNN models. Human experts give importance Region of Interests (ROI) to make decisions whereas CNN use posterior probability functions to make decisions^9, 10^.

With the success of CNN and its effectiveness in facial expression recognition, attention mechanism was widely adopted in many studies which learn intermediate attention maps and apply element-wise product on both source and attention maps to assign weights all features based on its importance i.e. whether features are primary or secondary. Weight assignation on features helped to improve the recognition results which could be better than the results of CNN models^11^. Generally, CNN with attention mechanism was proposed to obscured area of the face image and to recognize most discriminating un-obscured area of the image.CNN with Attention mechanism is either path based (pACNN) which focuses on partial facial patches or global local based (gACN12N) which combines local and global representation at the patch level and the image level^12^.

Long Short Term Memory (LSTM) can be combined with CNN to learn from the image sequences using time dependencies which helps in forming a memory with gating units to control the accumulation speed of information and deals with disappearance of gradients. In addition, LSTM is effective in processing time series and managing long term dependencies, and best feature combination. Though, LSTM learns correlation between sequence data, it fails to achieve the same in long-term^13, 14^. To resolve this problem of LSTM, Bi-LSTM was introduced which performs back propagation and forward propagation with more accuracy. Each input training sequence is given to Bi-LSTM in both the directions to successfully separate the recurrent nets^14^.

The main contributions of this research work are as follows:

- In the first stage, original images of datasets are reconstructed into 64*64 pixels by either increasing the size or decreasing the size of the image using Histogram of Oriented Gradients (HOG) method.
- Then, a Deep CNN is combined with Binary Short Long Term Memory to convolute and reiterate the images with necessary information which includes both primary and secondary features of facial images.
- With obtained facial features, Self attention and Channel attention is performed on both spatial and channel domains to extract both local and global information from the output of Bi-LSTM feature maps for creating better feature vector maps using cross-function fusion.
- Linear and Piecewise Cubic Polynomial (PCP) activation functions are used to speed up the operational processes in order to reduce the computation power and complexity of the system model.
- Finally, softmax classifier is used to classify the images into seven different classes based on the expression present in the input facial images.

The novelty or the highlights of this paper is as follows: DCNN is opted as it has dynamic convolution layers to tackle variable size inputs in terms of length (sequence of images), width and height. With facial landmarks that we have adopted in our previous works will help to achieve improved accuracy by learning complex features automatically during training stage of DCNN, which also avoid the challenges off over fitting and difficulties in processing or dealing with subtle expressions or occlusions present in the large data considered in this study. This study have considered only 64 primary and secondary landmarks of facial images which significantly reduce over-fitting issue and computational requirements as the landmark portions of the image is learned instead of considering the whole image like SWIN and vision transformer based models. These models will attend the image as a whole at once which might increase computation power and cost. Though, transformer based models are effective in capturing global dependencies with long range interactions, our study do consider the image as a whole, but it aims to extract facial landmarks from the input images. So, transformers are not considered in this research study^15^. The input image is fed through forward layer and doing the reverse in backward layer is the special metric of Bi-LSTM to not miss any of the facial image target information both locally and globally from the input images. Dual attention mechanism is placed to recheck the validity of gathered information and by adopting this technique rich information can be obtained through validating the acquired facial expression information by reducing the information loss, thus improving accuracy of recognition rate.

In our previous work, we have used ReLU activation function^15^ which could suffer slow running speed with Deeper CNN and also have neuron inactivation problems. To tackle these difficulties faced by traditional CNN methods, we have adopted piecewise cubic polynomial function (PCP) as an activation function that can enhance Interactive Learning Mechanism (ILP) for feature fusion, to speed up the process with reduced computation cost and complexity to overcome the issues faced by sigmoid, tanh and ReLU activation functions such as exponential slow down in operation speed and ReLU which suffers with neuron death issues and high computational cost issues. In many scenarios, softmax has delivered better classification when hybrid or integrated approaches were proposed, which is far better than classifiers like Support Vector Machine (SVM) and Multi-Lib SVM. Hence, the proposed model has both authority and novelty in achieving high performance not only in terms of accuracy metric, but with some other metrics also used in this paper.

This research paper is organized as follows: Sect. Related work, briefly discusses about the existing state of the art approaches related to our research and also draws the drawbacks and limitations of those approaches. Section 3, presents the motivation of this research work which is followed by the proposed work In Sect. 4 and Sect. 5 presents the experimentation results of this research work and comparative analysis with the existing approaches. Finally, Sect. 6 concludes the research with future work ideas.

## Related work

To avoid collusions and unexpected incidents, road safety must be ensured and to recognize the Facial Expression of the driver is very much essential as it directly connects with the scenario. It is necessary to determine that whether the individual is expelling the truth or not. In the world of Artificial Intelligence, FER technology is growing among the manufacturers and customers to identify the suspects through their photos by detecting their facial expressions. Recent developments on Artificial Intelligence algorithms have given hope to public that computers can understand their emotions with more accuracy^16, 17^.

In^18^, authors have proposed a novel Harmonious Representation Learning (HRL) to learn Landmark Guided graph Message Propagation (LGMP) and also used generic matching metrics to determine spatially invariant feature selection on dataset images. This study has considered three dataset images namely, SFEW 2.0, RAF-DB and CK+. Style Aggregated Network (SAN) was adopted to identify landmarks on SFEW 2.0 input images since no landmark is provided for SFEW 2.0 whereas RAF-DB and CK + images has landmarks on its images. In^19^, complex number based data augmentation was proposed which obtain new representation from the original images, then fuses the original images and converts the obtained images into complex numbers using simple combination. Linear combination was used on all training images and space classification is done using kernel function. Finally, collaboration representation based classification was used to predict the facial expression of all images on ORL and Yale datasets. This approach has achieved high recognition rates than other state of the art methods.

In^20^, authors have proposed Generative Adversarial Network (GAN) based multi-angle facial expression recognition method which uses depth regression network to identify the key points of the original image, thus feature extraction would be easier. After feature extraction, the image is given as input to GAN which comprises of encoder, decoder and a skip connection. The output of GAN is forwarded to CNN for classification and learning. To enhance recognition accuracy four loss weight functions were used: resistance, recognition, center and content loss. This approach was implemented on PIE and CFP datasets. In^21^, the authors have proposed an approach to tackle challenges on recognizing emotions from dynamic sequences of facial images in real time using CNN and Bi-LSTM. Spatial feature of images are extracted using VGG-19 skeleton and these features are fed as input to Bi-LSTM to extract spatiotemporal features of time series in both directions. This approach was implemented on CK + and in house datasets and hold-out-cross validation technique was employed to verify the proposed approach. The results showed that this approach has achieved 0.92% accuracy on CK + and 0.84% on in-house datasets.

In^22^, authors have proposed pyramid Fourier frequency conversion method to improve recognition on non-frontal and blurred images since discrete Fourier transform is very much effective frequency band of correct expression larger than incorrect expression of the same person’s face. The proposed method adjust incorrect expressions in multi scales and eliminated them out of band-pass CNN operations i.e. eliminated completely, and the correct expressions are reserved for effective recognition. In^23^, facial deflection and large parameters are addressed using Depth-wise Separable Convolution (DSC) along with DenseNet that improves the standard convolution. The deflection issue was addressed using a GAN based poster normalization model with two local discriminators which concentrates on nose, mouth, eyes and eyebrows of a facial image. The proposed approach was experimented on FER2013 and KDEF datasets and the results were better than other state of the art approaches.

In^24^, the authors have merged multilayer features of a lightweight convolutional neural network to address the issue of poor real-time recognition and larger number of parameters. Initially, a light weighted convolution network was designed using an improved inverted residual network with pooling, 1*1 convolutions and global average pooling. This approach was validated on RAF-DB and Affect Net datasets which produced accuracy of 85.49% and 57.70% respectively. In^25^, authors have proposed a multi-scale efficient channel attention mechanism to extract more abundant features of an expression and attention mechanism module improves response of the proposed model for extracting important information. Both shallow and deep layer information were fused by using cross-connection to avoid information loss of shallow layer.

In^26^, authors have proposed a GAN based non-frontal face recognition model which employs two channel generator and auto-coding network to separately encode the angle information of a facial image. A multi-discriminator mechanism was used to extract local parts of facial image to ensure the clarity to the maximum extent. The recognition results were obtained using FaceNet and MTCNN on multi-PIE and CFP datasets and the recognition accuracy of non-frontal images was improved by 1% in compared with VGG-FACE, TP-CNN and HPN. In^27^, authors have proposed DCNN with Binary Attention Mechanism (BAM) where original pixel data characteristics of an image used to train the model and employed HOG to prepare data and to overcome over-fitting issue dropout, batch normalization and L2 regularization methods were used. The proposed model was compared with CNN, IBH, GWO, LSTM, but the DCNN-BAM has outperformed these approaches in various metrics including accuracy.

In^28^, authors have proposed RCL-Net to recognize wild facial expressions using attention mechanism and Local Binary Pattern (LBP) feature fusion, which consists of residual attention branch (ResNet-CBAM) and LBP extraction branch with RCL-Net. By merging these branches, local feature facial information from facial images were obtained from both spatial and channel dimensions to construct a residual attention classification model. Then, locally improved residual network attention model was used to extract texture information which enhances recognition accuracy of the proposed model. The proposed approach was experimented on various datasets like FER2013, FERPLUS, CK + and RAF-DB, and the results demonstrated that the proposed model produced superior generalization accuracy capability of 74.23% than recent existing methods. This model has achieved 5.94%, 2.69%, 7.37% and 5.20% improvements on FER2013, FERPLUS, CK + and RAF-DB datasets respectively than Baseline model and ResNet combined with CBAM models.

In^29^, authors have proposed a deep neural network with attention mechanism which not only focus on key attributes of the facial images, also overcomes the limitation of CNN’s inability to capture the long-ranged global features. The model has max-pooling layer following each convolution layer and rectified linear unit (ReLU) activation function and two fully connected layers create a spatial transformer that performs localization. The spatial transformer segments the distorted data and concentrate on important parts of the input image on the attended region. This study has employed affine transformation technique for warping the input to output while training was done using a optimization loss function with Adam Optimizer operates using Stochastic Gradient Descent Approach. The summation of regularization and classification loss computes the loss function. The proposed model was implemented in FER2013 and CK + datasets, but no comparisons were done with other state of the approaches.

In general, using AI techniques or algorithms will require more computation power with high complexity. In^30^, authors have proposed Custom Lightweight CNN based model (CLCM) based on MobileNetV2 and the model was implemented on FER2013, RAF-DB and AffectNet datasets. This model was smaller model when considering parameters, yet it has achieved accuracy of 63% on FER2013, 84% on RAF-DB, 54% on AffectNet. In^31^, Custom-built CNN was proposed to improve accuracy and optimize computational efficiency and the proposed model was evaluated on Indian Spontaneous Expression Dataset (ISED) and Indian Semi-acted Facial Expression Database (iSAFE). This model has adopted manual extraction, thus training time was reduced and the accuracy was improved by 11.14% on ISED and 4.72% on iSAFE. The results were compared with ResNet-50 where 0.27% improved accuracy and 0.24% improved accuracy for proposed approach achieved.

In^32^, author have proposed ConvNet model which employed Local Binary Pattern LBP) and region based Oriented FAST and rotated BRIEF (ORB) to extract features and these features are fed to CNN for classification through fusing. To evaluate the performance of this model, FER2013, JAFFE and CK + datasets were selected and generalization techniques were applied. This approach has attained 92.05% accuracy on JAFFE, 91.01% on FER2013 and 98.13% on CK + datasets. In^33^, CNN based Bi-LSTM model was proposed which performs data augmentation to prevent over-fitting and improve performance of the proposed approach. With data augmentation, this model has achieved 99.43% accuracy on CK + dataset detecting 7 emotions. Min-max normalization was used to normalize images and features were extracted to build feature map or activation map.

In^34^, an attention mechanism based dual path FER network model was proposed with two paths: first to obtain the emotional feature maps from the input images and secondly to obtain noise feature maps at the background of the images. This model reduce the background noise features that exist in the emotional features maps by subtracting the feature maps output from these two paths, by extracting key features the ability f both these paths are improved with the help of attention mechanism. This model has achieved the test accuracy on FER2013 dataset images with 71.47% while testing. In^35^, Dual Direction Attention Mixed Feature Network (DDAMFN) was proposed with lightweight and robustness characteristics for FER using two components: firstly, a Mixed Feature Network (MFN) which acts as a backbone, secondly a head called Dual-Direction Attention Network (DDA) which captures long range dependencies by generating two orientations based attention maps. In addition, attention loss mechanism was adapted with DDA sing different heads to enhance the accuracy by focusing on distinctive areas of the facial input images. The proposed model was experimented on datasets like AffectNet, RAF-DB and FERPlus to evaluate the detection accuracy of FER and results were compared with existing approaches like Point Light Displays (PLD), Region Attention Network (RAN), Squeeze and Excitation 50 (SeNet50), RAN-VGG16, Self-Cure Network (SCN), Kernelized Tree Networks (KTN) and Transfer Learning-FER (TransFER) approaches where the proposed model DDAMFN delivered superior performance that other approaches in detection accuracy percentage.

In^36^, CBAM-Global Efficient Channel Attention-ResNet (C-G-ECA-R) based strong mechanism was proposed for FER where important facial feature expressions were extracted by embedding channel attention module before residual network and spatial attention module after residual network. The facial information loss is reduced and key features are retained during the extraction by adding Global-Efficient Channel Attention to the residual network module. The experimentation was done on JAFFE and CK + datasets and the C-G-ECA—R model has achieved the accuracy of 98.98% and 97.65% respectively.

In^37^, cross-fusion dual attention network was proposed with three stages: first, to obtain global features after refining the local features using cross-fusion grouped dual attention mechanism, secondly, a piece-wise cubic polynomial construction method with three dimensions used to minimize the computation overhead, improve recognition ability and overcoming slow running and inactivation neuron problems. Finally, to suppress the redundant information a closed-loop operation was performed between self-attention distillation and residual connection that can also helps in improving the generalization performance of the proposed approach. The model was experimented on three datasets, namely, RAF-DB, FERPlus and AfectNet and achieved the accuracy of 92.78%, 92.02% and 63.58% respectively.

The following section of this paper discusses our motivation of this research paper.

## Motivation of this research

Facial expression recognition research works have been expanding over the years both in academia and research. FER has become an interesting research topic due to its versatility in recognizing expression of a face both in real time, static or analyzing videos. Though, there has much research works have been done on FER, it imposes many challenges to the aspirants since the datasets or videos can consist of frontal images, non frontal images with different scenes, poses, weather, lighting, dark and so on. In addition, the aspirants need to address issues like over-fitting, number of parameters, improving accuracy with adequate and most suitable models and algorithms. In the recent past, many researchers have opted for Generative Adversarial Networks (GAN) to reduce the false recognition rate to ensure the designed model and its performance is iterated for further improvement to attain expected results and performance. Most of the deep learning methods were considered in FER such as CNN, ANN, DCNN, GAN, LSTM, Bi-LSTM, ResNet and etc. In the same way, attention mechanism module was also used to improve the accuracy in many research works. Yet, there still exist a need to add most appropriate and combined approaches with efficient validation and feature extraction methods to bring out better recognition rate which can significantly improve the prediction accuracy percentage. The above mentioned points along with the learning from the literature work have encouraged the researchers to opt for FER in this paper. In this paper, we propose a hybrid model for FER with DCNN-Bi-LSTM with dual attention mechanism. Data augmentation, batch normalization is considered along with Interactive Learning Mechanism (ILM) for validation and softmax for classification of expressions in our proposed approach.

The novelty of the proposed approach is to avoid both over-fitting and under-fitting issues of training the DCNN model with the considered datasets and also to consider the datasets with enhanced image quality features by adapting HOG which allows the proposed model to improve the accuracy of recognizing the facial emotion features from these HOG generated output images. In addition to this, the proposed model was designed with the aim of reducing the computational overhead and complexity issues through some modifications in DCNN convolution layers and bringing in ILI along with softmax classifier. The following section of this paper introduces the proposed model and presents the discussions in detail.

## Proposed methodology

From the literature study and researcher’s observation on Facial Expression Recognition, it is evident that basic CNN-BiLSTM fails to perform well for non frontal and occluded images, also images with different poses and angles. Thus, we propose a Deep Convolutional Network Layer with Binary Long Short Term Memory by adopting Dual Attention Mechanism module for better control over recognizing facial expression of a person’s image. It is important to extract both global and local information, importantly primary parameters that define face and what it expresses such as eyebrow, nose, mouth, teeth and so on. Along with these models and modules, some other methods such as HOG, cross-fusion and PCP are also adopted for better feature extraction, normalization and validation to ensure that the prediction accuracy is better than that of existing state-of-the approaches.

### Datasets

We have considered seven facial expressions in the datasets we have opted for implementing this proposed model, namely, “happy, sad, fear, disgust, angry, sad and surprise” and this study has considered 3 data sets such as FER 2013, CK + and RAF-DB. These datasets evaluates and verifies the recognition accuracy, computational complexity and also some other metrics such as recall, F1 score, sensitivity and selectivity. These results are used to compare the proposed approach’s performance with some other existing state of the art approaches for performance evaluation purposes.

Cohn Kanade + dataset^38^ consists of 327 images with aforementioned 7 emotions (6 basic emotions and neutral) whereas FER 2013 dataset^39^ consist of 35,887 images of different scenes, poses, angles, occlusion, unclear, blurred and trembling scenarios (6 basic emotions and neutral). Real World Affective Faces Database (RAF-DB)^40^ which has a total of 29,673 facial image expressions of thousands of independent individuals. All these images have great variability in terms of sex, age, ethnicity, occlusion, head pose, lighting and hair etc. This dataset contains both gray scale and RGB images with 100*100 pixels. Among 29,673 images 15,500 are neutral images and remaining images express any of the other 6 emotions. Japanese Female Facial Expression (JAFFE) dataset^41,42^ have 213 images captured from 10 individual females with different universal facial expressions.

**Table 1:** Summary of datasets used for this work.

Dataset	uni-modal/multi modal	Modalities	No. of Images	No. of Citations	No. of Participants
CK+	Uni-modal	Images	327	123	4091
FER 2013	Uni-modal	Images	28,709	85	3589
RAF-DB	Uni-modal	Images	29,672	92	40
JAFFE	Uni-modal	Images	213	436	10

**Fig. 1:**
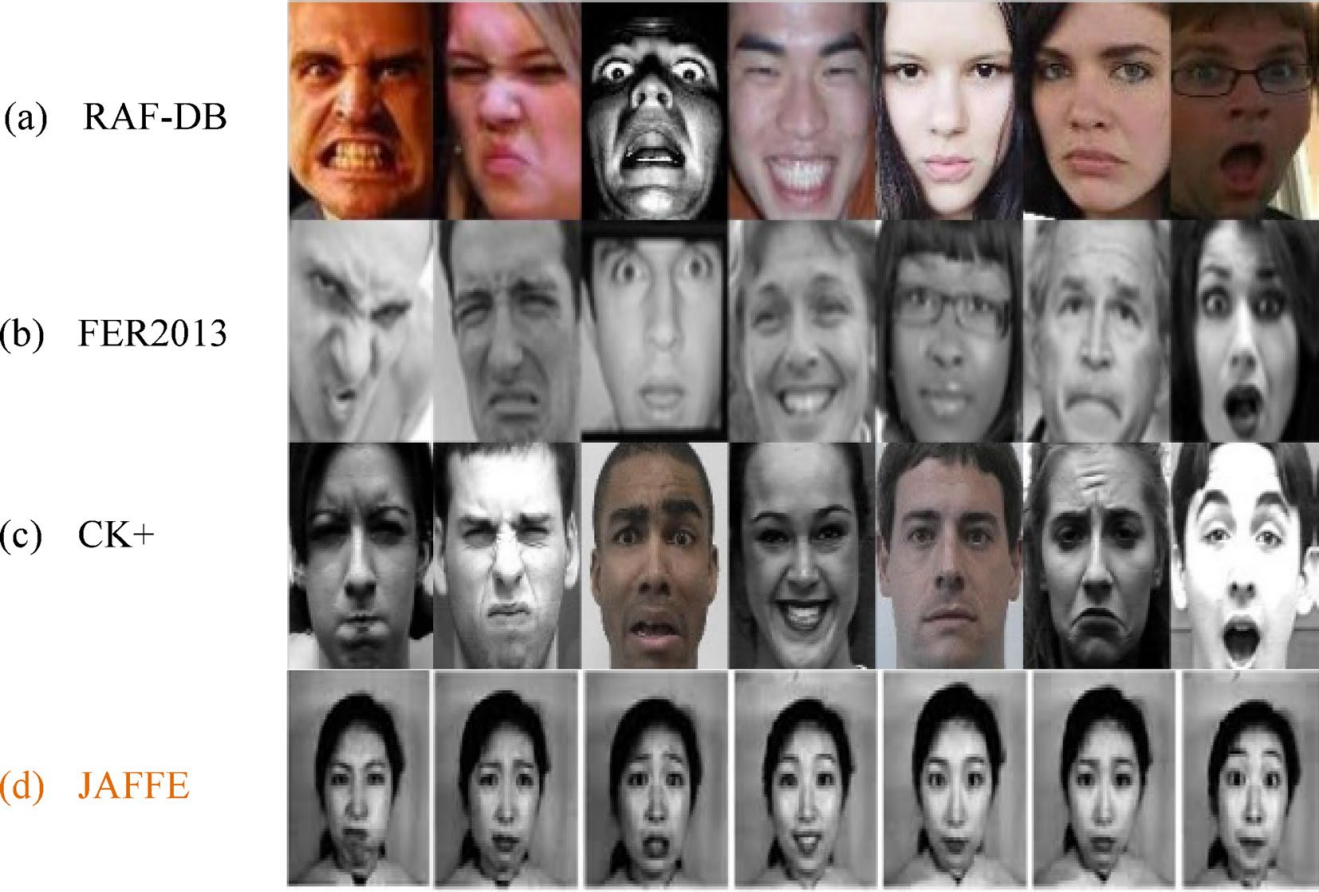
Facial Expression images of studied datasets (7 emotions)^30^.

Table 1 presents the concise summary of datasets considered for this study and Fig. 1 presents sample images with 7 different facial expressions from each datasets of this study. Once the input image is obtained, the face is identified and cropped from that image where its pixel is normalized into 64*64 pixels.

### Feature extraction and normalization

Histogram of Oriented Gradients is the most widely used feature descriptor method for face and image detection to classify image expression classes by converting the original input image into $$\:\left(Width*Height*Channels\right)$$ feature vector maps with user defined length $$\:n$$. HOG performs better than flat region methods as it is best in checking edges and corners of images with high intensity which results in extracting vital information from the input images. HOG comptes both horizontal and vertical component of the gradients magnitude and direction for each individual pixel (64*64) and creates a 9-bin histogram which determines shifts in the data. For 3*3 pixel blocks, the gradientss of the image is calculated using the following formula.

1 $$\:{GT}_{X}(row,column)=IG\left(row,column+1\right)-IG\left(row,coloumn-1\right)$$

2 $$\:{GT}_{Y}(row,column)=IG\left(row-1,column\right)-IG\left(row+1,coloumn\right)$$

where $\:IG$ =image and $\:{GT}_{X},{GT}_{Y}\:$represents horiontal and vertical gradients of an image. Once $\:{GT}_{X}\:and{GT}_{Y}$ is calculated, the magnitude and angle for each pixel is computed using,

3 $$\:Maginitude\:\left(\mu\:\right)=\sqrt{{GT}_{X}^{2}+{GT}_{Y}^{2}\:}$$

4 $$\:Angle\:\left(\theta\:\right)=\left|{tan}^{-1}\left({GT}_{Y}/{GT}_{X}\right)\right|$$

Following this, blocks are formed by divinding gradient metrices into 8*8 cells and 9-bin histogram is created for each blocks and each bin has angle range of 20 degree.

5 $$\:No.of\:bins=9\left(ranges\:from\:{0}^{0}\:to\:{180}^{0}\right)$$

6 $$\:step\:size=\left(\varDelta\:\theta\:\right)={180}^{0}\:\:/\:No.of\:bins={20}^{0}$$

Bin boundaries for each bin and center values for each bin is calculated, then the histogram equation and L2 normalization of each block is written as,

7 $$\:{H}_{bi}=\left[{b}_{1},{b}_{2},{b}_{3},\dots\:\dots\:.,{b}_{36}\right]\:and\:{H}_{bi}\leftarrow\:\frac{{H}_{bi}}{\sqrt{{\parallel{H}_{bi}\parallel}^{2}+\epsilon\:}}$$

where $\:\epsilon\:$ is small value used to avoid the zero division error by adding it to the square of $\:{H}_{bi}$. In order to complete the normalization, the value of $\:k$ is calclated, then it is sed for normalization. The following equation is used,

8 $$\:k=\sqrt{{b}_{1}^{2}+{b}_{2}^{2}+{b}_{3}^{2}+..\dots\:.+{b}_{36}^{2}}\:\mathrm{a}\mathrm{n}\mathrm{d}\:{H}_{bi}=\left[\left(\frac{{b}_{1}}{k}\right),\left(\frac{{b}_{2}}{k}\right),\left(\frac{{b}_{3}}{k}\right),\dots\:..,\left(\frac{{b}_{36}}{k}\right)\right]$$

Histogram of Oriented Gradients (HOG) is used to normalize the image for better data preparation and reduce the impact of lighting on image and other environmental varilable that are likely to disrupt FER performance.

### Deep convolutional neural networks (DCNN)

**Fig. 2:**
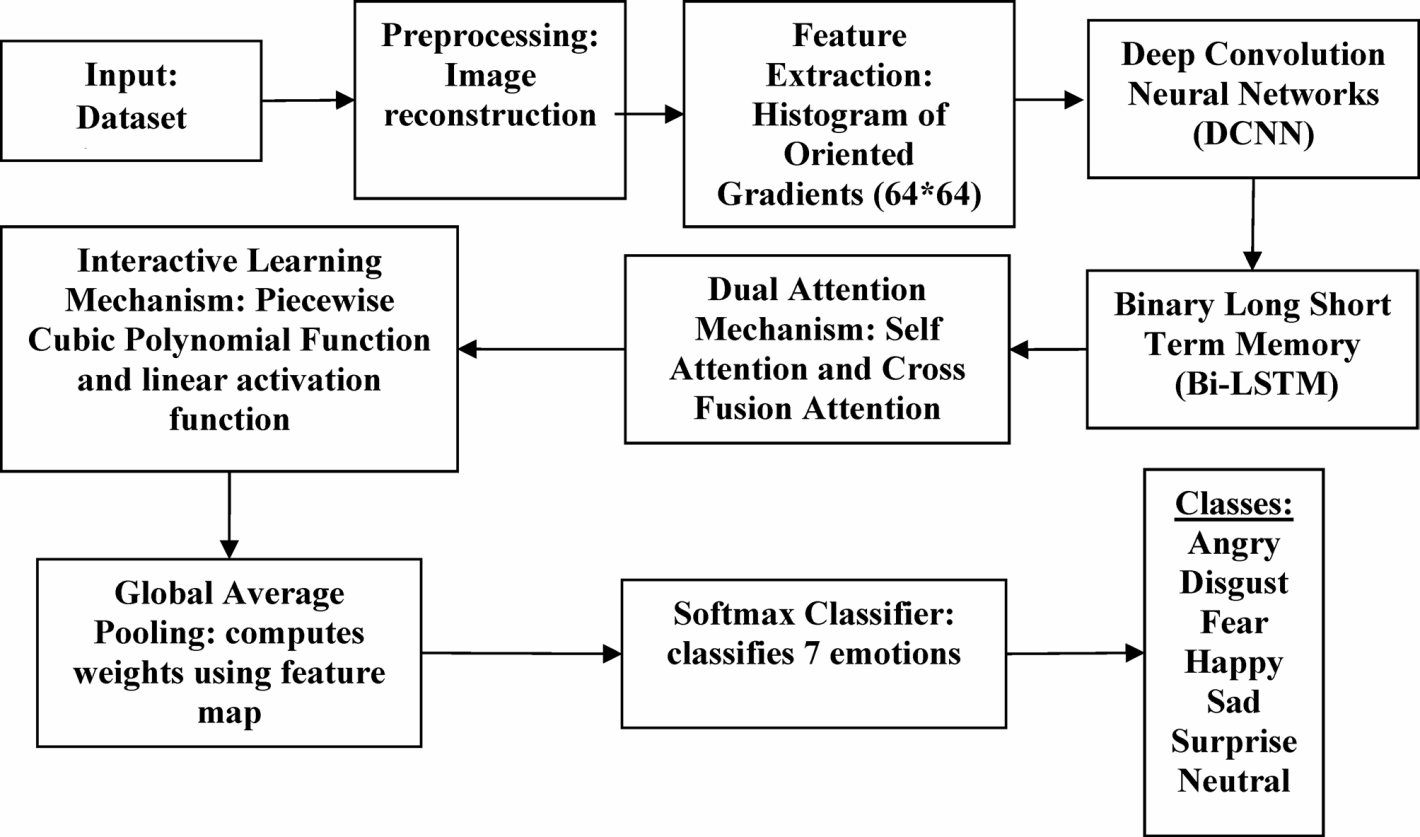
Overall architecture of proposed model.

Though CNN has achieved significant results in FER, DCNN is adopted due its effectiveness in dropout and batch normalization features which partially drops some neurons since DCNN gathers more detailed characterization of data from large datasets as well. This helps DCNN to deliver better recognition results through having ‘convolution’ and ‘max-pooling’ using two max-pooling $\:({S}_{1}\:and\:{S}_{2)\:}$and three convolutions$\:({C}_{1},{C}_{2},{C}_{3)}$. Figure 2 represents the overall architecture of proposed approach.

With DCNN, the sliding window one at a time for convolution to compute the top input layer using the following equation,

9 $$\:{J}_{y}^{c}=\theta\:\left(\sum\:_{x=1}^{{P}_{y}^{C-1}}{CK}_{x,y}\otimes\:{i}_{x}^{C-1}+{S}_{y}^{C}\right),y=\mathrm{1,2},3,\dots\:N$$

where current layer is $\:C$ and previous layer is$\:C-1$, $\:{i}_{x}^{C-1}$ is a layer before current layer’s feature graph, $\:{J}_{y}^{c}$ is the feature graph of the current layer. Bias feature graph is taken as $\:{S}_{y}^{C}$, no. of features maps retained at present is $\:N$, activation function is represented as $\:\theta\:(.)$ and $\:{P}_{y}^{C-1}$ denotes the layer which is currently connected to layer 1. Finally, $\:{S}_{y}^{C}$ is used to minimize learning parameters and speed up the experiment. Since the pixel sixe used in this approach is 64*64 and nucleus of convolution is 5*5, the feature map obtained through this model is 52*42. In total, 128 feature maps are created. The pooling layers function is presented in the following equation,

10 $$\:{J}_{C}^{y}=\theta\:\left({\beta\:}_{y}^{C}Down\left({J}_{C}^{y-1}\right)+c\right)$$

Figure 2 represented by Overall architecture of proposed model above, Where the first and current layer’s feature maps are represented as $\:{J}_{C}^{y}$ and $\:{J}_{C}^{y-1}$ and $\:\theta\:(.)$denotes down sampling function. $\:{\beta\:}_{y}^{C}$ represents activation function which was discussed earlier with additive and multiplicative bias. The fully connected or complete connection layer converts a two dimensional array into a single dimensional one using the following equation,

11 $$\:{OT}_{C,S}\left(i\right)=\theta\:\left({e}^{P}i+S\right)$$

where $\:{OT}_{C,S}\left(i\right)$ denotes the neurons output value, in which I represents the Eigen vector of input neurons, $\:e$ is the weighting factor and $\:S$ denotes bias function.

All the primary features of proposed DCNN model parameters on undertaken dataset images are presented in Table 2.

**Table 2:** DCNN features used in this approach.

Layers	Inversion stack	Nucleus of convolution	Sample window	Fully connected level	Fully linked Neurons	Range of Emotions
*C*_*1*_	32	5*5 images	–	–	–	7
*C*_*2*_	64	5*5 images	–	–	–	7
*C*_*3*_	128	5*5 images	–	–	–	7
*S*_*1*_	–	–	2*2 Images	–	–	7
*S*_*2*_	–	–	2*2 Images	300 cells	–	7

### Bi-LSTM architecture

**Fig. 3:**
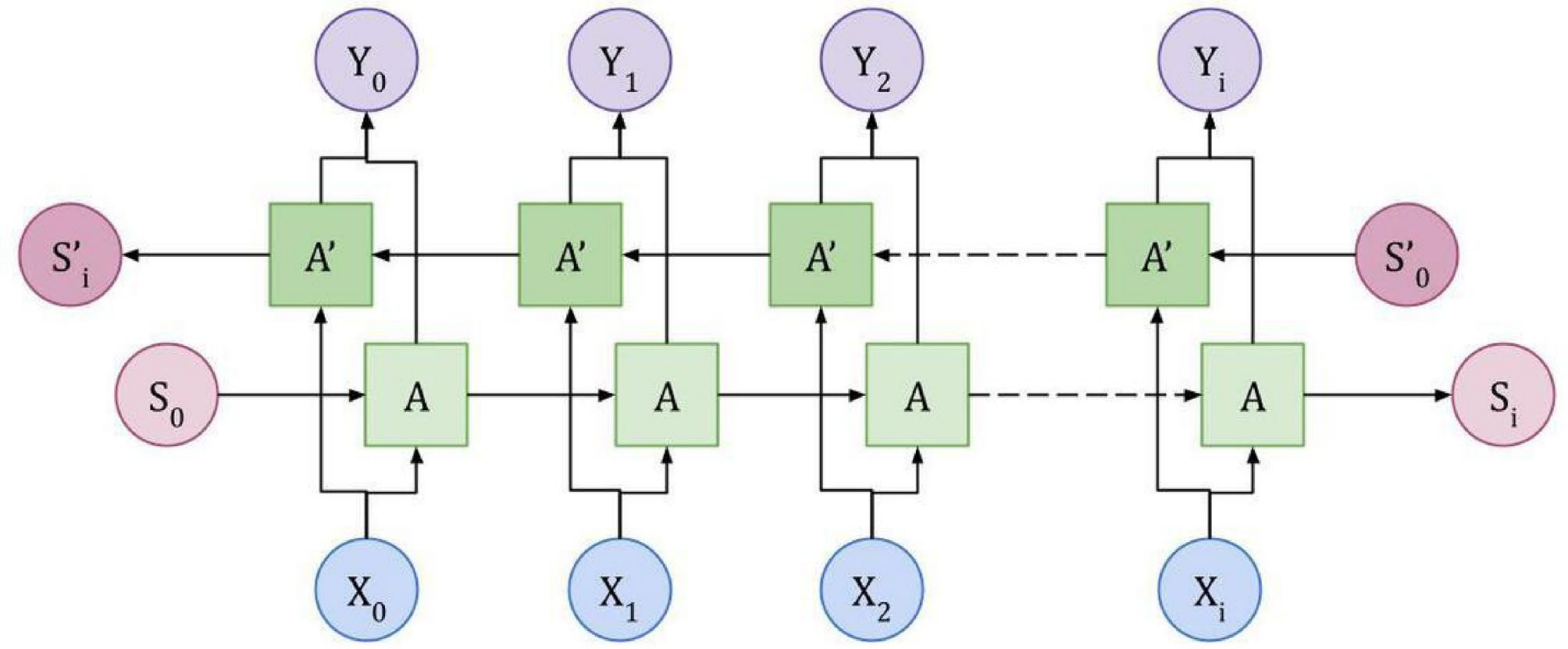
Bidirectional-LSTM Layer Architecture.

Bi-LSTM architecture consists of ‘gates’ (internal mechanisms) and few linear interactions with two layers i.e. forward and backward that helps in determining what data from an image needs to be retained and what data needs to be discarded. Figure 3 represents the Bi-LSTM architecture adopted in this paper Bi-LSTM is very much effective in dealing with images that are taken at different time periods and angles as it mainly extracts time series of information from the input images, such information is trustworthy in obtaining time series feature vectors that can be fed to the Dual Attention Mechanism module of our proposed approach. Bi-LSTM model used in this paper is presented in Fig. 3 where $\:{X}_{i}$ denotes the input token and $\:{Y}_{i}$ denotes the output token which is the combination of $\:A\:and{A}^{{\prime\:}}$ LSTM nodes. In general, Bi-LSTM is the combination of two unidirectional LSTM that process the image sequence in both forward (gets tokens as it is) and backward directions (gets tokens in reverse order). Probabilities of both these layers are combined to obtain the final probability vector map. This can be expressed in the following equation.

12 $$\:{PV}_{T}={PV}_{T}^{FD}+{PV}_{T}^{BD}$$

In which, $\:{PV}_{T}\:$represents the final probability vector, $\:{PV}_{T}^{FD}$ represents the probability vector of forward layer and $\:{PV}_{T}^{BD}$ represents the probability vector of backward layer.

**Fig. 4:**
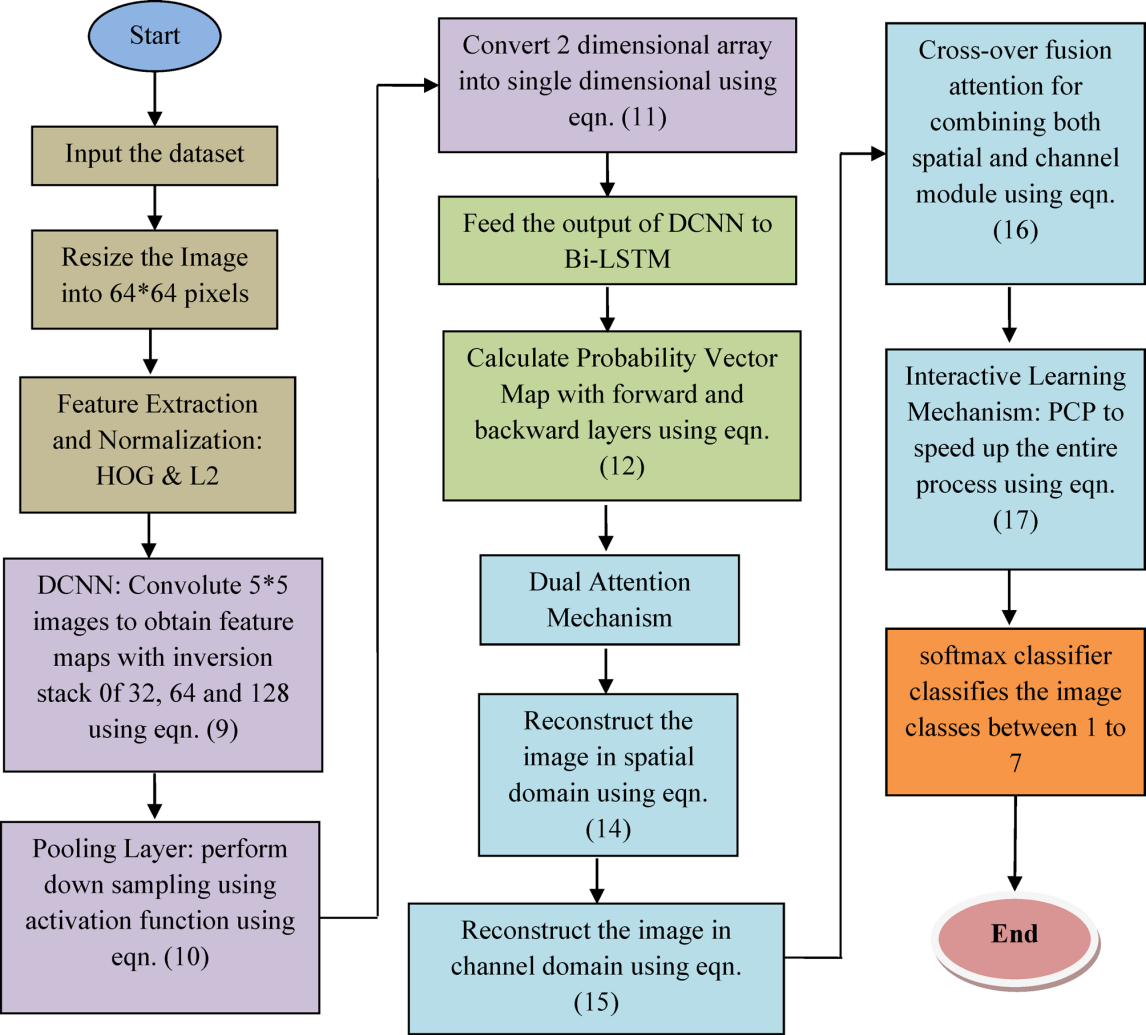
Working methodology of the proposed Model.

The context relationship of the sequence for all images of the undertaken data sets such as FER2013, CK + and RAF-DB can be automatically generated by extracting sequence features from the dataset images. These features provide rich information of images to the network model and also increase the recognition rate and accuracy of facial image expression recognition. The entire flow of the proposed model is presented in Fig. 4.

### Dual attention mechanism (DAM)

In the field of FER, identifying global features and relationships of an object is difficult and challengeable, and this cannot be attained by simple encoder and decoder structures which fail to establish such relationships within an image features^27^. Architectures like RNN heavily depends on long term memorization of the output, whereas Binary Attention Mechanism (BAM) and Convolution Block Attention Module (CBAM) employs simple stacking convolutions to create spatial attention maps which fails to capture global information. This paper has adopted Dual Attention Mechanism (DAM) which uses self attention module to get global information directly.

**Fig. 5:**
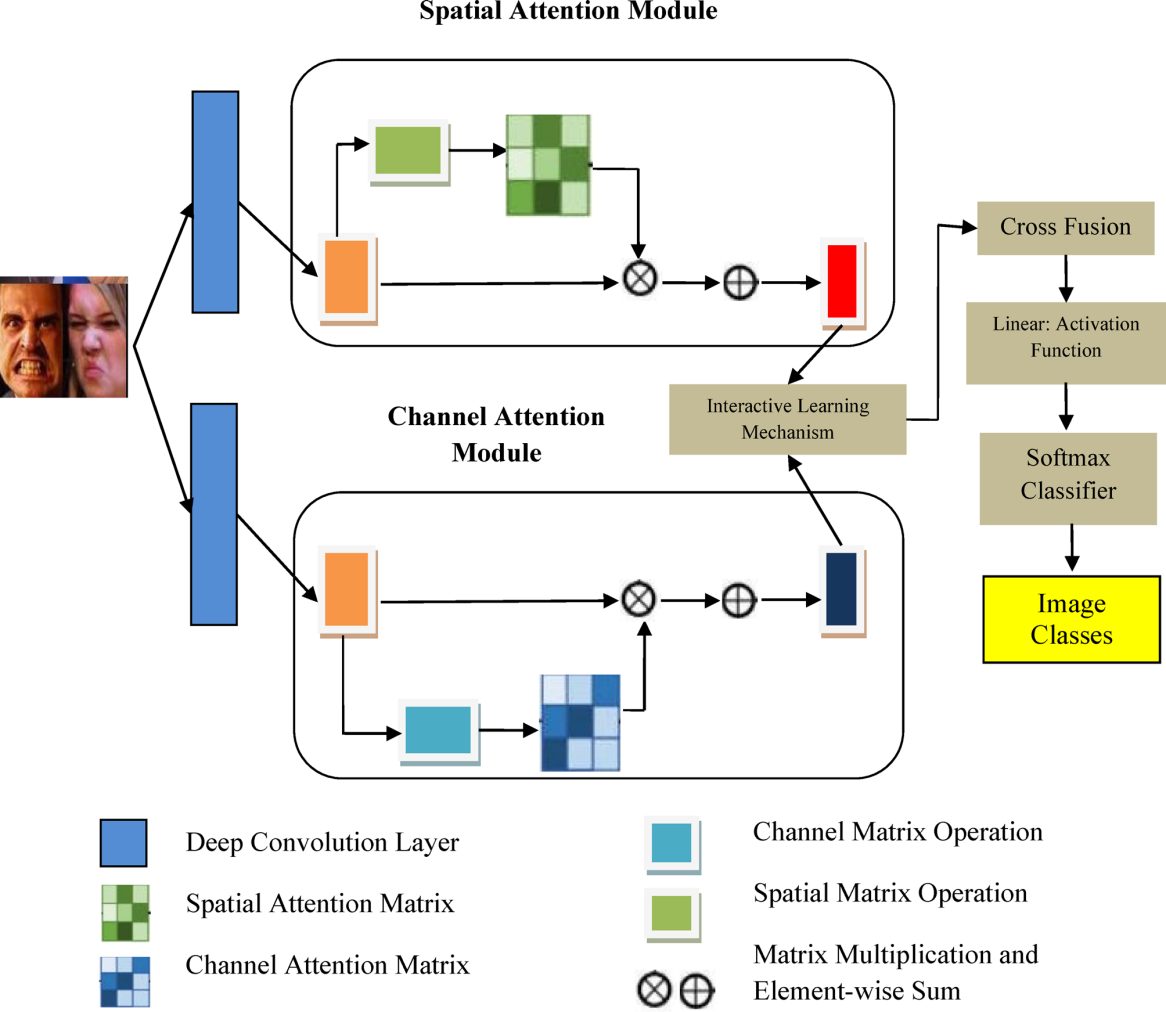
Structure of Dual Attention Mechanism.

In this paper, we propose a dual attention mechanism (represented in Fig. 5) which combines self-attention mechanism of both channel and spatial dimension and both are operated in parallel. Group based learning method was added with channel attention to complement each other dimensions. This also helps to acquire both local and global information that improves FER abilities. In spatial dimension, reconstructed images were formed into $\:{R}_{g}$ groups and each group is divided into $\:{R}_{p}$ non-overlapping patches using Sliding Window approach and these patches will be grouped again later. The size of reconstructed image depends on original image size and desired resolution of the reconstructed image.

#### Algorithm 1

**Self attention mechanism in spatial dimension**.

**Input: Feature map of the CNN-BiLSTM reconstructed image**.

**Output: Attention Feature Map**.

Step 1: Input reconstructed grayscale image features of DCNN-Bi-LSTM.

Q = Input * sq//query (H*W*C) -> (H*W*C_q).

K = Input *sk//key (H*W*C-> (H*W*C_k).

V = Input *sv//value (H*W*C-> (H*W*C_v).

Step 2: Divide the image into $\:{R}_{g}$ groups and $\:{R}_{p}$ non-overlapping patches using Sliding Window approach

Step 3: Compute size of each patch (in pixels) with $\:Width*Height$ and $\:{R}_{g}$ groups

Step 4: for each $\:{R}_{p}$ compute pixel values

Step 5: $\:{P}_{pv}=\left(W/{R}_{g}\right)*\left(H/{R}_{g}\right)$//pixel values for each patch

//Attention Weights computation.

Step 6: $$\:{AT}_{wt}=\left(H*W\right)*\left(H*W\right)-Nomalized\:attetnion\:scores$$

Step 7: $$\:{AT}_{fm}={AT}_{wt}*v$$

//Residual connection and Normalization.

Step 8: $$\:Residual\:\left({AT}_{fm}\right)={AT}_{fm}+Input$$

Step 9: $\:{OAT}_{fm}=normalize\left(Residual\:\left({AT}_{fm}\right)\right)$//output attention feature map

Step 10: Overall process in spatial dimension is given in Eq. (14)

Step 11: The result is $\:{Q}_{1},{K}_{1},{V}_{1},$…$\:\:{Q}_{{R}_{g}},{K}_{{R}_{g}},{V}_{{R}_{g}}\in\:{R}^{{R}_{g}*C}$

Let’s consider, the dimensions of reconstructed image $\:Width*Height$ and groups $\:{R}_{g}$, the pixel size for each patch is determined by using the following,

13 $$\:\left(\frac{Width}{{R}_{g}}\right)*\left(\frac{Height}{{R}_{p}}\right)$$

The entire process of Attention Mechanism on spatial dimension can be written as,

14 $$\:{AM}_{S}\left(Q,K,V\right)=Concat({AM}_{1}^{*}\left({Q}_{1},{K}_{1},{V}_{1}\right),\dots\:\dots\:..{AM}_{{R}_{g}}^{*}({Q}_{{R}_{g}},{K}_{{R}_{g}},{V}_{{R}_{g}}\:\left)\right)$$

Concatenation function is used to fuse two different features extracted by two different methods in order to obtain a combined unique image. the In recent study^33, 34, 35^, the researchers have reconstructed the original image using linear transformation of the input image by dividing spatial dimension into patches and positional coding, as a result the obtained image can be represented by $\:{RC}^{P*C}$ where $\:P\:and\:C$ denotes patches and channels. In such case, unique two dimensional information of an input image is completely destroyed and recovering lost information is very much difficult challenge. To overcome this issue, dual attention mechanism was used in this study, so self-attention result of this study on spatial dimension is $\:{AM}_{1}^{*}\left({Q}_{1},{K}_{1},{V}_{1}\right),\dots\:\dots\:..{AM}_{{R}_{g}}^{*}({Q}_{{R}_{g}},{K}_{{R}_{g}},{V}_{{R}_{g}})$ and $\:{Q}_{1},{K}_{1},{V}_{1},$…$\:\:{Q}_{{R}_{g}},{K}_{{R}_{g}},{V}_{{R}_{g}}\in\:{R}^{{R}_{g}*C}$.

#### Algorithm 2

**Self attention mechanism in channel dimension and cross fusion**.

**Input: Feature maps and reduced features vectors**.

**Output: classification off expression label**.

Step 1: Input reconstructed grayscale image in spatial dimension.

Step 2: Divide the image into $\:{G}_{w}$ groups and $\:{CH}_{w}$ channels

Step 3: The value of C is $\:C={G}_{w}$*$\:{CH}_{w}$

Step 4: Overall process in channel dimension is given in eqn. (15)

Step 5: The result is $\:{Q}_{1},{K}_{1},{V}_{1},$…$\:\:{Q}_{{R}_{g}},{K}_{{R}_{g}},{V}_{{R}_{g}}\in\:{R}^{{R}_{g}*C}$

//Cross-fusion attention on spatial dimension.

Step 6: Map ($\:{AM}_{S}^{l}\in\:{R}^{P*C})\leftrightarrow\:{Q}_{S}^{l}\:and\:{V}_{S}^{l}$//two linear transformations

$$\:\mathrm{S}\mathrm{t}\mathrm{e}\mathrm{p}\:7:\:{RD}_{fv}=\:\mathrm{M}\mathrm{a}\mathrm{p}\:({AM}_{S}^{l}\in\:{R}^{P*C})\leftrightarrow\:{Q}_{S}^{l}\:and\:{V}_{S}^{l}$$ 

//Cross-fusion attention on channel dimension.

Step 8: Map $\:({AM}_{c}^{l}\in\:{R}^{P*C})\leftrightarrow\:{K}_{C}^{l}$//2^nd^ linear transformation.

Step 9: Forward $\:{Q}_{S}^{l},{K}_{C}^{l},{and\:V}_{S}^{l}$ to the spatial dimension of next layer for self attentions

//Final Attention feature map.

Step 10: The attention feature map $\:{FAT}_{fm}$= $$\:\left\{\begin{array}{c}{AM}_{S}^{l+1}={AM}_{S}({Q}_{S}^{l},{K}_{C}^{l},{V}_{S}^{l})\\\:{AM}_{C}^{l+1}={AM}_{C}({Q}_{C}^{l},{K}_{S}^{l},{V}_{C}^{l})\end{array}\:\:\:\right.$$

Step 11: Train softmax classifier//input: fused features and output: Predicted emotion class.

Step 12: $\:F{{EX}_{label}=softmax(FAT}_{fm})$//Facial expression label classification

In channel attention, reconstructed image is divided into $\:{G}_{w}$ groups where each group consists of $\:{CH}_{w}$ channels i.e. $\:C={G}_{w}$*$\:{CH}_{w}$. The entire operational process on channel attention can be written as,

15 $$\:{AM}_{c}\left(Q,K,V\right)=Concat({AM}_{1}^{*}\left({Q}_{1},{K}_{1},{V}_{1}\right),\dots\:\dots\:.,\:{AM}_{{G}_{w}}^{*}({Q}_{{R}_{g}},{K}_{{R}_{g}},{V}_{{R}_{g}}\left)\right)$$

where $\:Q,K,V\in\:{R}^{P*C}$. From Eq. (14) and Eq. (15), it can be understood that both global and local interaction is established where channel-attention helps in obtaining global information presents on face and its surrounding environment whereas spatial attention is sensitive to key local locations such as lips, mouth, eye and etc. We employed cross-over method to make these two dimensions to cooperate together and operate in parallel.

Two linear transformations were used to map the output of the spatial dimension’s upper layer $\:{AM}_{S}^{l}\in\:{R}^{P*C}$ to two image matrixes $\:{Q}_{S}^{l}\:and\:{V}_{S}^{l}\:$. In the same way, the output of the channel dimension’s upper layer $\:{AM}_{c}^{l}\in\:{R}^{P*C}$ is mapped through linear transformation with image matrix $\:{K}_{C}^{l}$, which is send to the spatial dimension of the self attention’s next layer. This cross over fusion is performed using the following equation,

16 $$\:\left\{\begin{array}{c}{AM}_{S}^{l+1}={AM}_{S}({Q}_{S}^{l},{K}_{C}^{l},{V}_{S}^{l})\\\:{AM}_{C}^{l+1}={AM}_{C}({Q}_{C}^{l},{K}_{S}^{l},{V}_{C}^{l})\end{array}\right.$$

Figure 5 Structure of Dual Attention Mechanism below.

Unwanted interference can be introduced with excessive information while the attention window expands to the entire image. Since the study used facial landmarks for recognition the emotions from the facial images, self-attention distillation mechanism was used to avoid the redundant combinations of $\:K$ and $\:V$. An independent closed-loop operation was constructed with the interaction between self-attention distillation and residual connection that helps in reconstructing loss information as well.

#### Algorithm 3

**Self attention Distillation**.

Step 1: Reduce the excessive information of Keys $\:K$ and Values $\:V$ using facial landmarks

Step 2: Reduce scale of spatial and channel dimensions with $\:K$ and $\:V$

Step 3: Extract dominant features and form feature map.

Step 4: Use residual connection to fuse original V with self attention results.

Step 5: Construct independent closed-loop operation for training.

Step 6: For images $\:1\:to\:N$ do//from input data loader//from input data loader

$$\:{Tr}_{pn}={Tr}_{model}\left(batc{h}_{images}\right)$$

$$\:{St}_{pn}={St}_{model}(batc{h}_{images}$$ 

Step 7: Reconstruct lost information $\:Loss={Dn}_{loss}({St}_{pn},{Tr}_{pn})$

Step 8: Backpropogation.

Following this, We have constructed 2 convolution layers with 3 kernels in spatial dimension $\:{Conv}_{3}$, for mapping the channel number from high to low dimension is performed at first layer (extract potential dominant features), in the second layer the same operation was performed reversely, from low to high to ensure same dimension as original input image. Number of patches in keys and values were reduced using the max pooling layer $\:MxPL$. The complete process is represented as,

17 $$\:\left\{\begin{array}{c}{DN}_{s}\left(K\right)=MxPL({Conv}_{3}+\left({Conv}_{3}-\left(Keys\right)\right))\\\:{DN}_{s}\left(V\right)=MxPL({Conv}_{3}+\left({Conv}_{3}-\left(Values\right)\right))\end{array}\:\right.$$

To reduce the number of channels in single group, $\:{Conv}_{1}$ was used and to learn more reliable features and enhance the connection between channels $\:{Conv}_{3}$ was used in channel dimension. We also avoided using max pooling in here to acquire important global information, thus the patch numbers in each group is unchanged. This process is represented as,

18 $$\:\left\{\begin{array}{c}{DN}_{c}\left(K\right)={Conv}_{3}+\left({Conv}_{1}\left(Keys\right)\right)\\\:{DN}_{c}\left(V\right)={Conv}_{3}+\left({Conv}_{1}\left(Values\right)\right)\end{array}\:\right.$$

We also used Piecewise Cubic Polynomial (PCP) as an activation function just to increase the calculation speed of Interactive Learning Mechanism (ILM). Unlike sigmoid and tanh which perform exponential operations that slows up the calculation speed whereas ReLU suffers with neuron death issue and non-differentiable points problems with the facial image. Thus, the overall ILP process can be written as,

19 $$\begin{aligned}\:ILM&\left({AM}_{S}\left(Q,K,V\right),{AM}_{C}\left(Q,K,V\right)\right)\\&=INIAM\left({AM}_{S}\left(Q,K,V\right),{AM}_{C}\left(Q,K,V\right)\right)+INIAM\left({AM}_{C}\left(Q,K,V\right),{AM}_{S}\left(Q,K,V\right)\right)\end{aligned}$$

where $\:INIAM$ represents the Interactive Information of Attention Mechanism module with PCP activation function. Softmax classifier will categorize the facial expressions of image at the final stage after ILP mechanism. Softmax is a generalized logistic function that takes as input a vector of scores $\:x\in\:{R}^{n}$and outputs a vector of probability $\:p\in\:{R}^{n}$ through a softmax function that is defined as follows,

20 $$\:{p}_{i}=\frac{{e}^{{x}_{i}}}{{\sum\:}_{j=1}^{n}{e}^{{x}_{i}}}$$

## Experimental results

The proposed approach was implemented in MATLAB 2021a with Windows 10 operating system and Intel i7 processor with 6GB RAM. As mentioned earlier, this study has considered three different types of datasets, namely, FER2013, CK + and RAF-DB. The batch size used in this model is 32 and 100 epochs used for the training along with a 0.001 Adam Optimizer and in order to save the history log Model Checkpoint and CSV_Logger callback are defined for the trained model. The study has considered all seven facial expressions present in the datasets and the facial expression category of each dataset is presented in Table 3. The computational cost of the proposed model was reduced significantly with the help of facial landmarks during the training, where the primary landmarks and secondary are assigned weights in teacher model and then the model size was reduced further reduced to potential features with high weights sing knowledge distillation. To reduce the number of operations, a separable convolution was used with depth-wise layer and point-wise layer without changing the convolution depth, therefore number of Floating Point Operations per Second (FLOPS) can be reduced. With this setup, the proposed model has the following rules for convolution and fully connected layers,

21 $$\:FLOPs=2*No.of\:Kernal*kernel\:Shape*Output\:shape$$

22 $$\:FLOPs=2*Input\:size*Output\:size$$

In a neural network model, one Multiply-Accumulate Computations (MAC) is equal to two FLOPS. The complexity of the model is determined by the length of the input volume size $\:I$, length of the filter $\:L$, the amount of zero padding $\:P$, the stride $\:S$ and the output size $\:O$ of the feature map along the dimension is,

23 $$\:O=\frac{I-F+{P}_{start}+{P}_{end}}{S}+1$$

where

24 $$\:{P}_{start}=\frac{S\lceil\frac{I}{S}\rceil-I+F-S}{2}\:\:\:,{P}_{start}\in\:\left[\left[0,F-1\right]\right]$$

25 $$\:{{P}_{end}=\frac{S\lceil\frac{I}{S}\rceil-I+F-S}{2},\:\:P}_{end}=F-1$$.

Therefore, the number off parameters at the convolution is $$\:\left(F*F*C+1\right).K$$and ‘0’ for pooling and for fully connected layer is $$\:{(N}_{in}+1)*{N}_{out}$$. In total, the model had 1,060,400 operations and the inference time is 1ms for 1 GFLOPS with the model size of 40 MB of image size 28*28*1 of grayscale image, 2 convolutions of 5 kernels of size (3*3), first FC of 128 neurons and final FC with 7 neurons:1 per emotion.

**Fig. 6:**
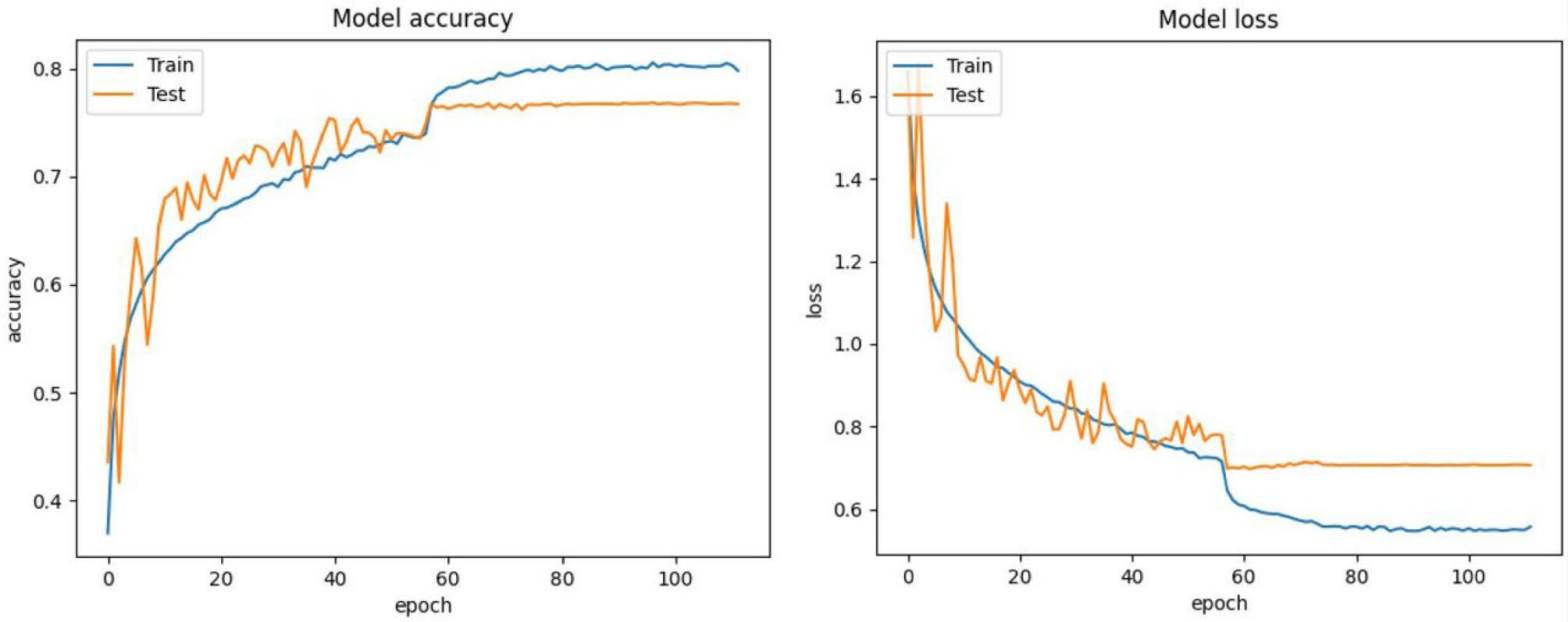
Model accuracy and loss of proposed approach on FER2013 dataset.

**Fig. 7:**
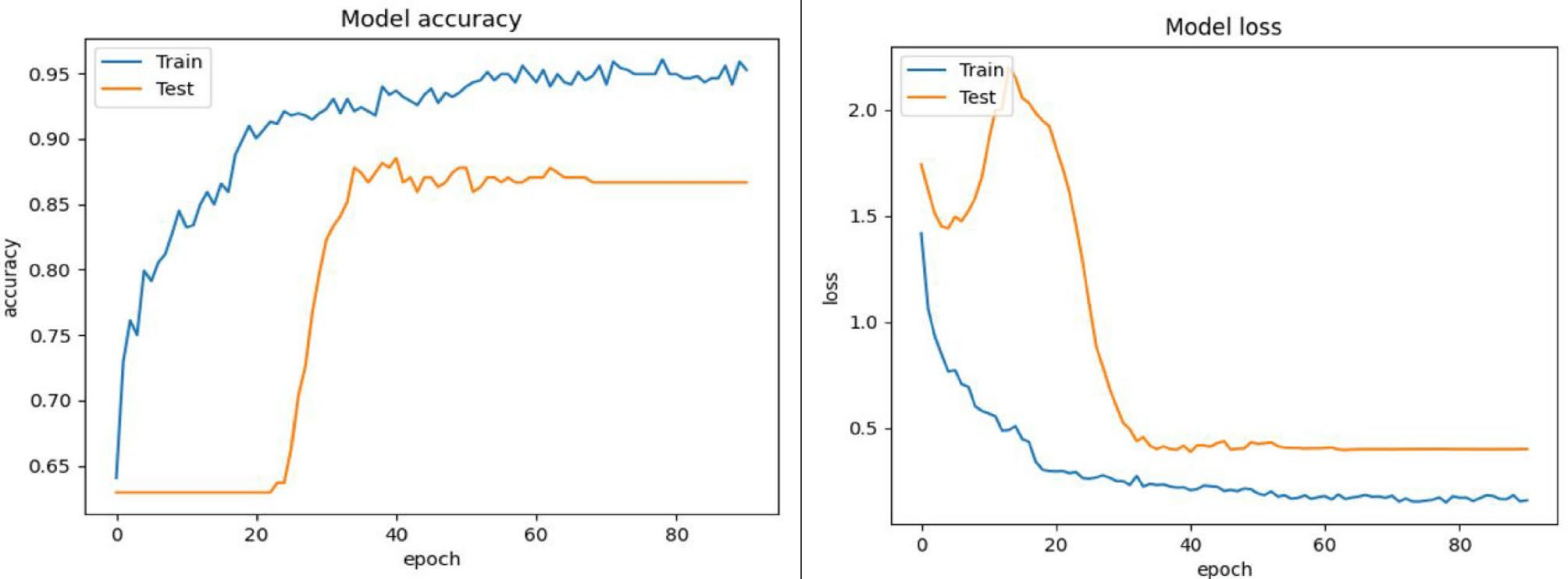
Model accuracy and loss of proposed approach on CK + dataset.

**Fig. 8:**
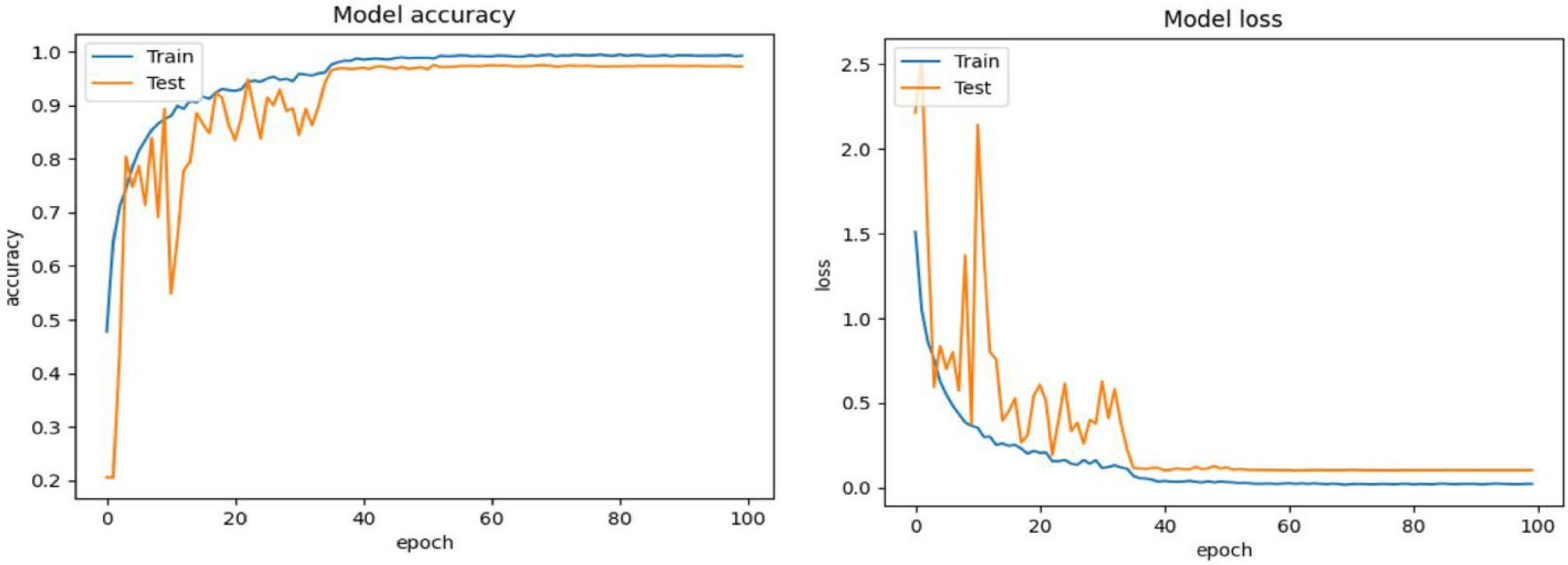
Model accuracy and loss of proposed approach on RAF-DB dataset.

Figures 6, 7 and 8 depicts the validation accuracy and loss ratio of our proposed model on different datasets like FER2013, CK+, RAF-DB. Estimating this helps to verify and validate the efficacy of proposed model, thus ensuring better FER rate and accuracy. This study have not considered AffectNet dataset which consists of more than 1 million images, thus training a model needs more computations power and complexity could be high. Due to laboratory hardware constraints and limitations, we were not able to implement the proposed model with AffectNet dataset. This study is also an enhancement of our previous research work where we have considered FER2013 dataset al.one^12^.

**Table 3:** Expression category distribution of datasets.

Training Set	Happy	Sad	Fear	Surprise	Disgust	Anger	Neutral	Total
FER 2013	4,077	5,543	5,190	4,077	541	4,967	6,275	30,670
CK+	69	28	25	83	59	45	593	902
RAF-DB	5,986	2,473	361	2,020	884	873	2,703	15,270
JAFFE	213	NA	NA	NA	NA	NA	NA	213

Since the female expressions of 10 women are considered in JAFFE dataset, the exact differentiation of emotions of expressions is not available. Thus, the total numbers of images are given and further emotion differentiation is not given as given to other datasets due to its unavailability. So, the expressions of emotions columns are left with not available (NA) and Table 3 presents the emotion distribution category of all images for each datasets^25^.

### Performance metrics

#### Accuracy

For any classification based research area, this metric is very much fundamental and common. Every research work might deal with plenty of issues and challenges, but accuracy metric is the most important one to figure out whether proposed model is effective than existing traditional approaches or not. In general, accuracy measures the overall correctness of the proposed model and it is measured with equation as follows,

26 $$\:Accuracy=\frac{Number\:of\:Correct\:Predictions}{Total\:Number\:of\:Predictions}$$

**Table 4:** Accuracy percentage comparisons.

Approaches	Various Datasets			
	FER2013	CK+	RAF-DB	JAFFE
NGO-BiLSTM [7]	81.23	93.12	92.45	91.02
ICNN-BiLSTM [16]	80.14	92.86	93.78	90.84
HCNN-LSTM [21]	81.14	89.82	91.43	90.27
Proposed	82.89	96.78	95.78	95.87

**Fig. 9:**
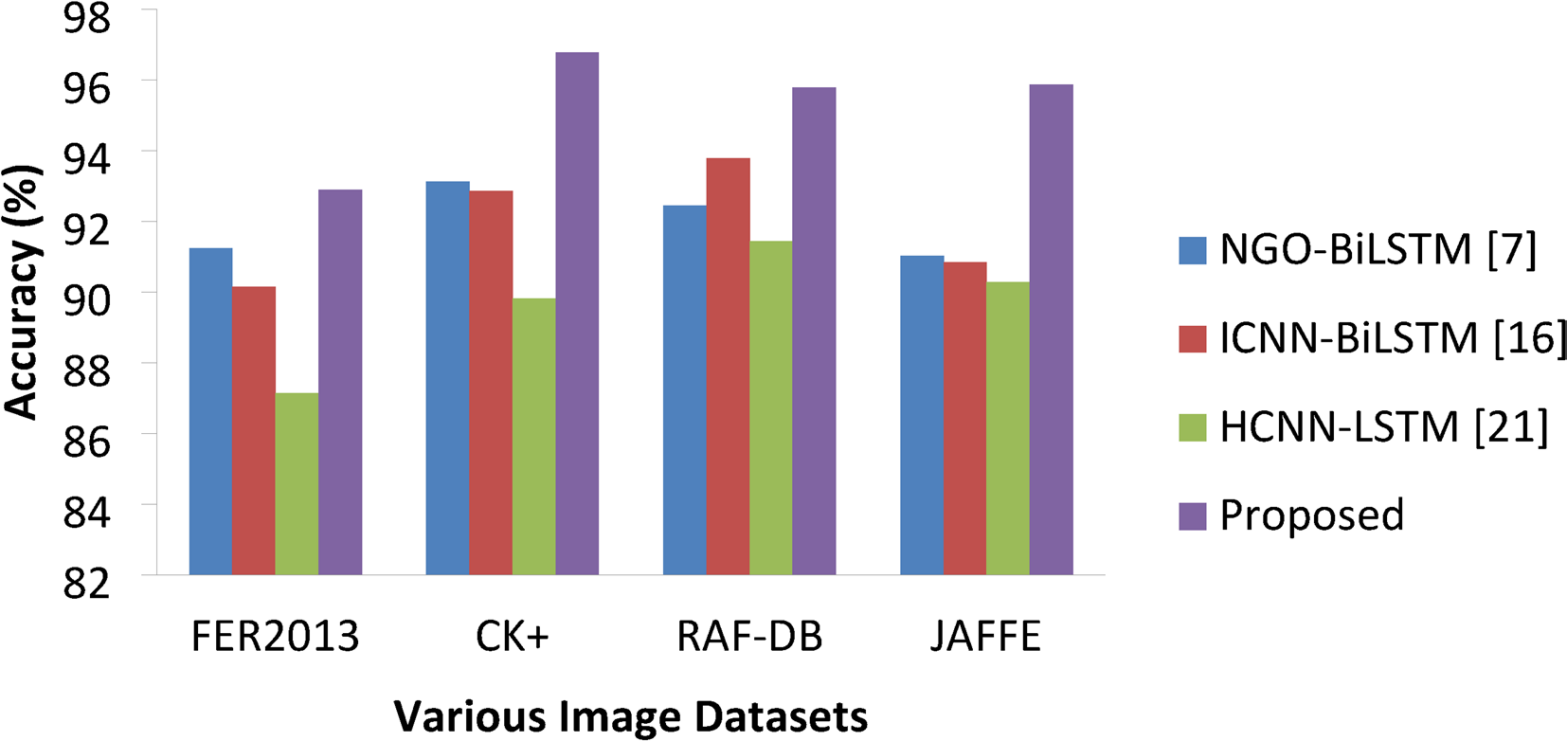
Accuracy percentages of various approaches.

Table 4 presents the % numbers for different approaches on different datasets and Fig. 9 represents the accuracy percentage achieved by various approaches on the datasets under study, in which the proposed DAM based DCNN-BiLSTM has achieved better accuracy than of other models like NGO-BiLSTM, ICNN-Bi-LSTM, HCNN-LSTM on all three datasets with percentages 82.89%, 96.78%, 95.78% and 95.87% on FER2013, CK+, RAF-DB and JAFFE respectively. NGO-Bi-LSTM offered the next best accuracy than other methods where HCNN-LSTM produced lower accuracy percentage than all other methods. The accuracy rate has significant variations depending on the datasets.

#### Specificity

It is a metric which is rarely used by the researchers as this metric falls under two specifications: two images not belong to the same expression or individual (TP) and those two images belongs to the same individual or expression (FP). A high specificity value indicates that the false positive rates are low. The equation can be written as,

27 $$\:Specificity=\frac{TN}{TN+FP}$$

**Table 5:** Specificity percentage comparisons.

Approaches	Various Datasets			
	FER2013	CK+	RAF-DB	JAFFE
NGO-BiLSTM [7]	89.87	92.17	92.11	91.83
ICNN-BiLSTM [16]	87.93	91.25	90.69	90.14
HCNN-LSTM [21]	82.54	89.84	87.24	89.87
Proposed	91.23	94.52	93.21	93.27

**Fig. 10:**
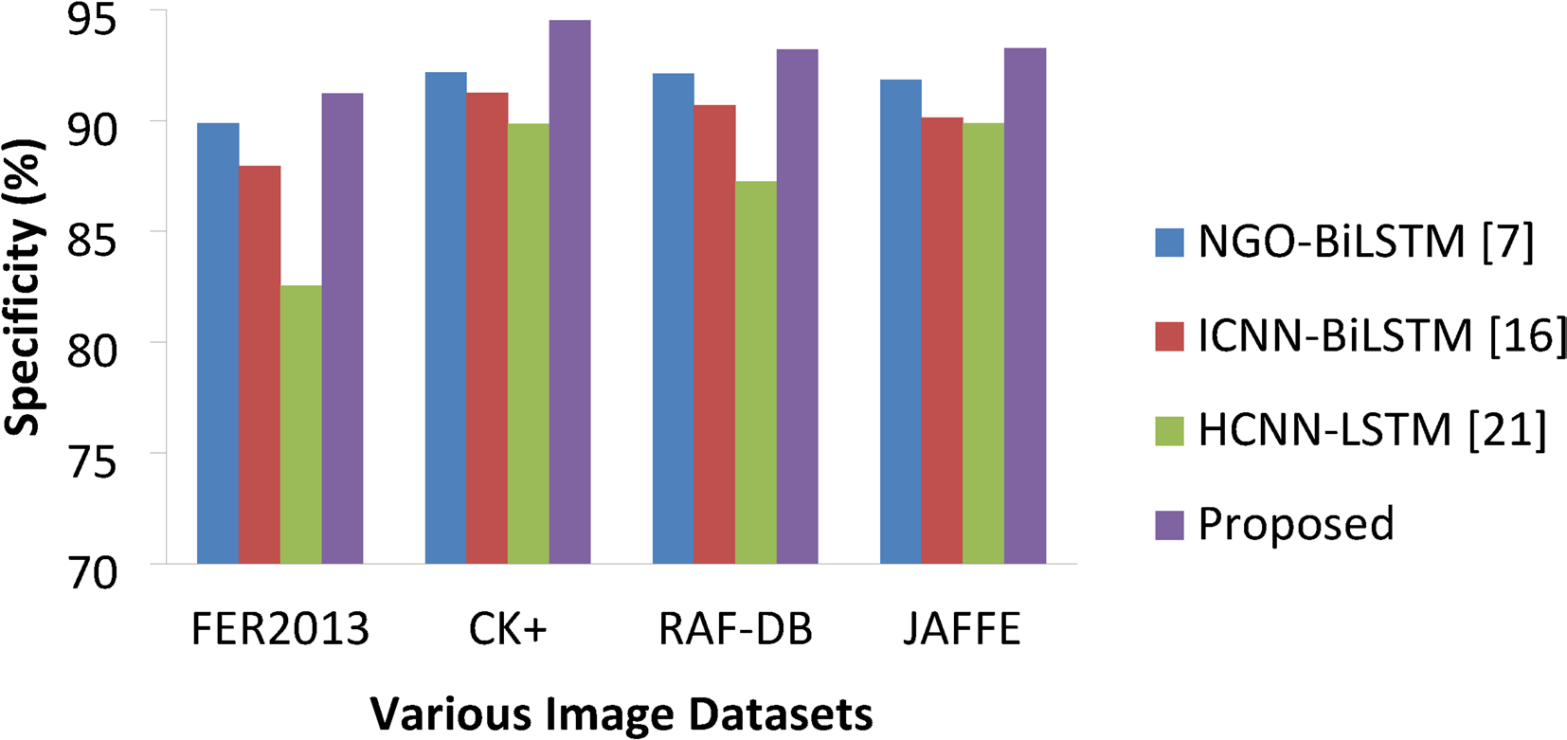
Specificity percentages of various approaches.

Table 5; Fig. 10 denotes the specificity values of various approaches on studied datasets. With this metric the proposed approach, NGO-BiLSTM and ICNN-BiLSTM models are very much competitive on CK + and RAF-DB datasets with small margins.

Yet, the proposed model was able to deliver improved specificity with 91.23%, 94.52%, 93.21% and 93.27% on each datasets which is 2.35% higher than NGO-BiLSTM and 3.23% higher than ICNN-Bi-LSTM on CK + dataset, and 1.11% higher than NGO-BiLSTM and 2.52% higher than ICNN-BiLSTM on RAF-DB datasets. On FER2013, the proposed model has achieved 1.36%, 3.3%, 8.69% higher than benchmarking methods respectively. On JAFFE, the DAB-DCNN-Bi-LSTM has achieved 1.44%, 3.13%, 3.4% higher specificity than of other methods. Overall, the proposed model produced better specificity than other methods.

#### Precision

This metric is used to measure positive identifications which are correct, yet high precision rate does not guarantee a low error rate. Like accuracy, precision also considered as an important metric for facial expression recognition. Following equation denotes precision calculation,

28 $$\:Precision=\frac{TP}{TP+FP}$$

**Table 6:** Precision percentage comparisons.

Approaches	Various Datasets			
	FER2013	CK+	RAF-DB	JAFFE
NGO-BiLSTM [7]	91.67	96.72	93.85	93.58
ICNN-BiLSTM [16]	93.22	94.74	94.67	94.21
HCNN-LSTM [21]	91.75	91.16	91.68	90.89
Proposed	95.27	97.54	95.63	95.26

**Fig. 11:**
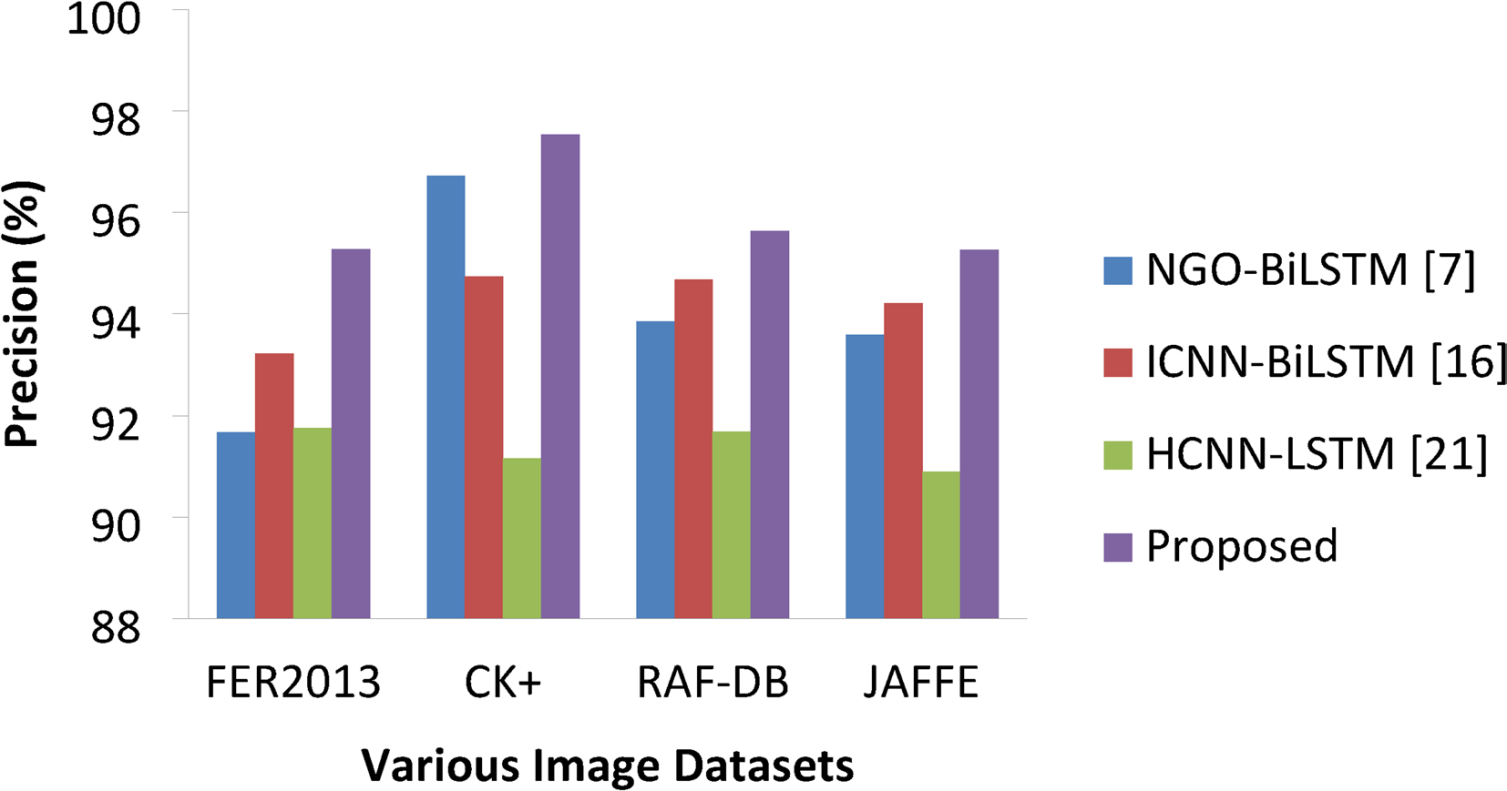
Precision percentages of various approaches.

As far as this metric is concerned, all the models have delivered better precision values with small differences in percentage (Fig. 11; Table 6). Still, the proposed model achieved 95.27%, 97.54%, 95.63% and 95.26% on FER 2013, CK+, RAF-DB and JAFFE respectively.

#### Recall or sensitivity

Recall metric is used to identify all the instances of a dataset that are relevant to recognize facial expression of an image. In other words, a higher recall value indicates that the model is very much correct in recognizing facial expressions accurately. The equation is given below,

29 $$\:Recall=\frac{TP}{TP+FN}$$

**Table 7:** Recall percentage Comparisons.

Approaches	Various Datasets			
	FER2013	CK+	RAF-DB	JAFFE
NGO-BiLSTM [7]	93.46	95.12	94.28	93.87
ICNN-BiLSTM [16]	92.92	93.97	92.36	92.58
HCNN-LSTM [21]	91.70	91.89	91.76	91.16
Proposed	94.18	96.67	95.79	95.22

**Fig. 12:**
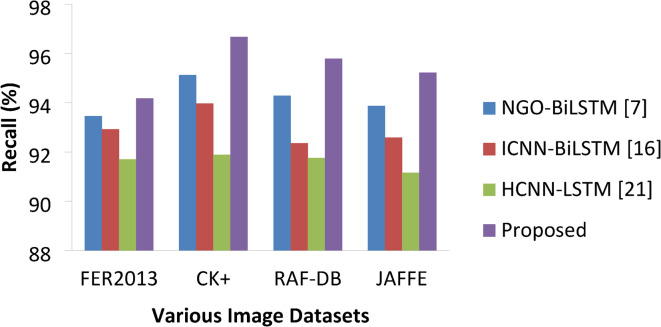
Recall percentages of various approaches.

The main objective of any study on FER would be to improve convolution network operation speed as it is directly proportional to the recall rate and the size of the image plays an important. As mentioned earlier, the images of the datasets are reconstructed into 64*64 pixels for all datasets to make the dataset image size uniform. Table 7 presents the values for various approaches on different datasets. Thus, DCNN speed can be further enhanced or increased. The proposed approach resulted with good recall values (Fig. 12) 94.18%, 96.67%, 95.79% and 95.22% the next best is attained by NGO-BiLSTM with values 93.46%, 95.12%, 94.28% and 93.87% on all three datasets FER2013, CK+, RAF-DB and JAFFE respectively.

#### F1 score

This is a crucial evaluation metric, especially, when dealing with imbalanced dataset as it is a measurement which balances both recall and precision. Among all the metrics of FER, F1 score is the most reliable metric that indicates the performance of the proposed model. The values of F1 score lies between 0 and 1 i.e.1 indicate impeccable precision and recall whereas 0 indicates possibly worst. It is measured with the following equation,

30 $$\:F1\:Score=2*\frac{Precision*Recall}{Precision+Recall}$$

**Fig. 13:**
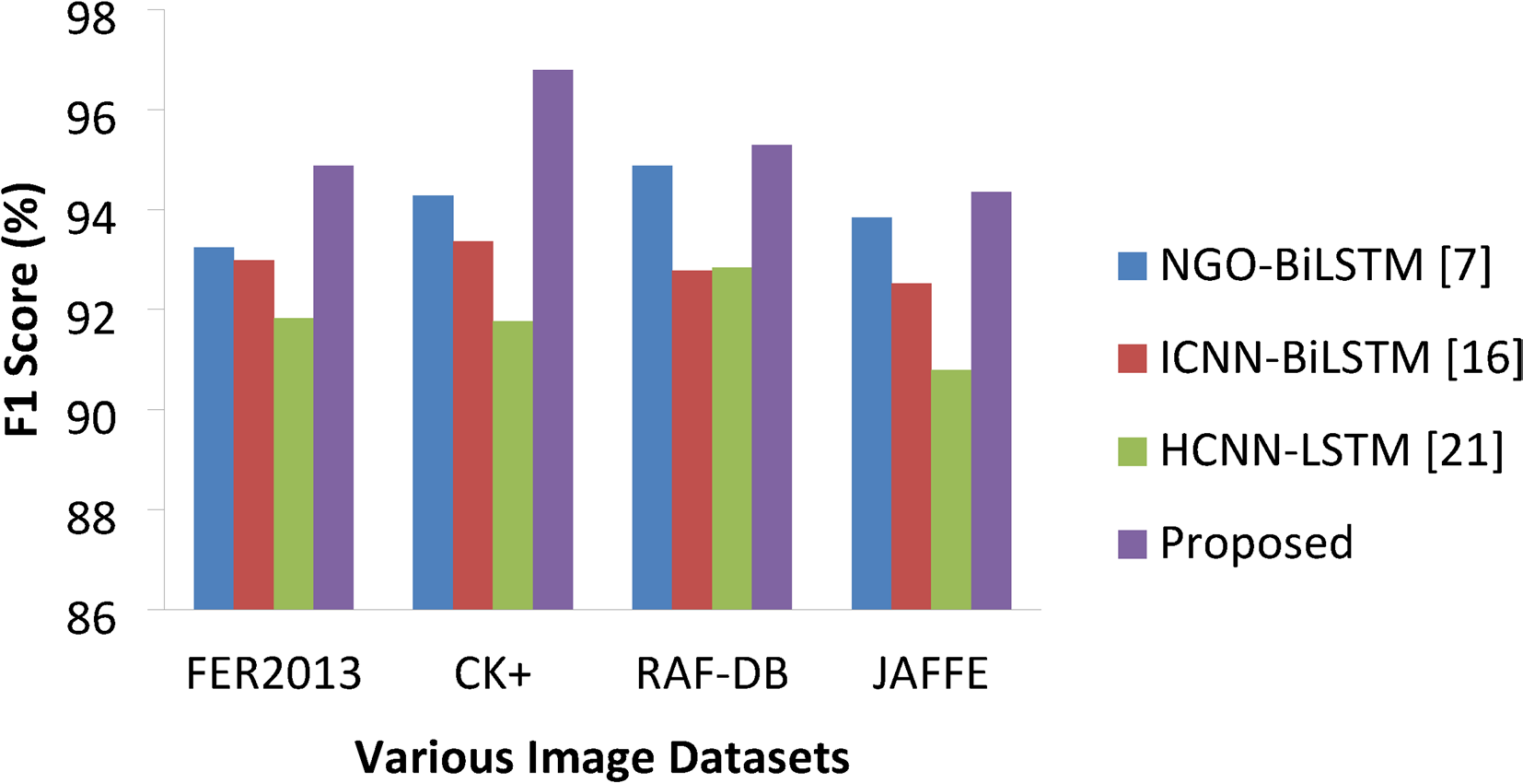
F1 Score percentages of various approaches.

**Table 8:** F1 score percentage Comparisons.

Approaches	Various Datasets			
	FER2013	CK+	RAF-DB	JAFFE
NGO-BiLSTM [7]	93.24	94.28	94.87	93.84
ICNN-BiLSTM [16]	92.98	93.36	92.78	92.52
HCNN-LSTM [21]	91.82	91.76	92.84	90.78
Proposed	94.87	96.79	95.29	94.35

Table 8 presents the recall percentages of various approaches on datasets under study. Since the reconstruction image with resizing (either increase or decrease), cropping helped the model to make the image measurements easier with suitable accuracy. Hence, our model has achieved (Fig. 13) 94.87%, 96.79%, 95.29% and 94.35% of F1-score on all four datasets which is 1.63% higher than the next best NGO-BiLSTM on FER 2013, 2.51% higher on CK + and 0.42% higher on RAF-DB and 0.51% higher on JAFFE. HCNN-LSTM resulted with lower numbers of 91.82%, 91.76%, 92.84% and 90.78% on all four datasets respectively.

#### Genuine acceptance or recognition rate (GAR)

GAR^43^ is the most significant metric for FER since it indicates the proportion valid inputs that are correctly recognized by the model with true validation. The ideal value for GAR should be high and it is expressed in the following equation,

31 $$\:GAR=\frac{No.of\:TP}{No.of\:TP+No.of\:FN}$$

**Table 9:** GAR Comparisons.

Approaches	Various Datasets			
	FER2013	CK+	RAF-DB	JAFFE
NGO-BiLSTM [7]	87.21	96.28	95.74	95.21
ICNN-BiLSTM [16]	84.35	94.78	92.49	94.67
HCNN-LSTM [21]	79.63	90.26	91.21	91.08
Proposed	88.57	97.23	96.87	96.32

#### False acceptance or recognition rate (FAR)

Basically, it is very much important to measure error rates as vital as acceptance rate for any research model. FAR is the value that the model wrongly identifies the expression and this metric also validates effectiveness of the model under study. It can be expressed as follows,

32 $$\:FRR=\frac{No.of\:False\:Acceptances}{Total\:\:No.of\:Attempts}*100$$

**Table 10:** FAR Comparisons.

Approaches	Various Datasets			
	FER2013	CK+	RAF-DB	JAFFE
NGO-BiLSTM [7]	9.12	4.21	5.47	4.32
ICNN-BiLSTM [16]	9.23	5.84	7.54	6.91
HCNN-LSTM [21]	11.23	7.35	8.41	7.82
Proposed	7.23	1.42	1.96	1.78

**Fig. 14:**
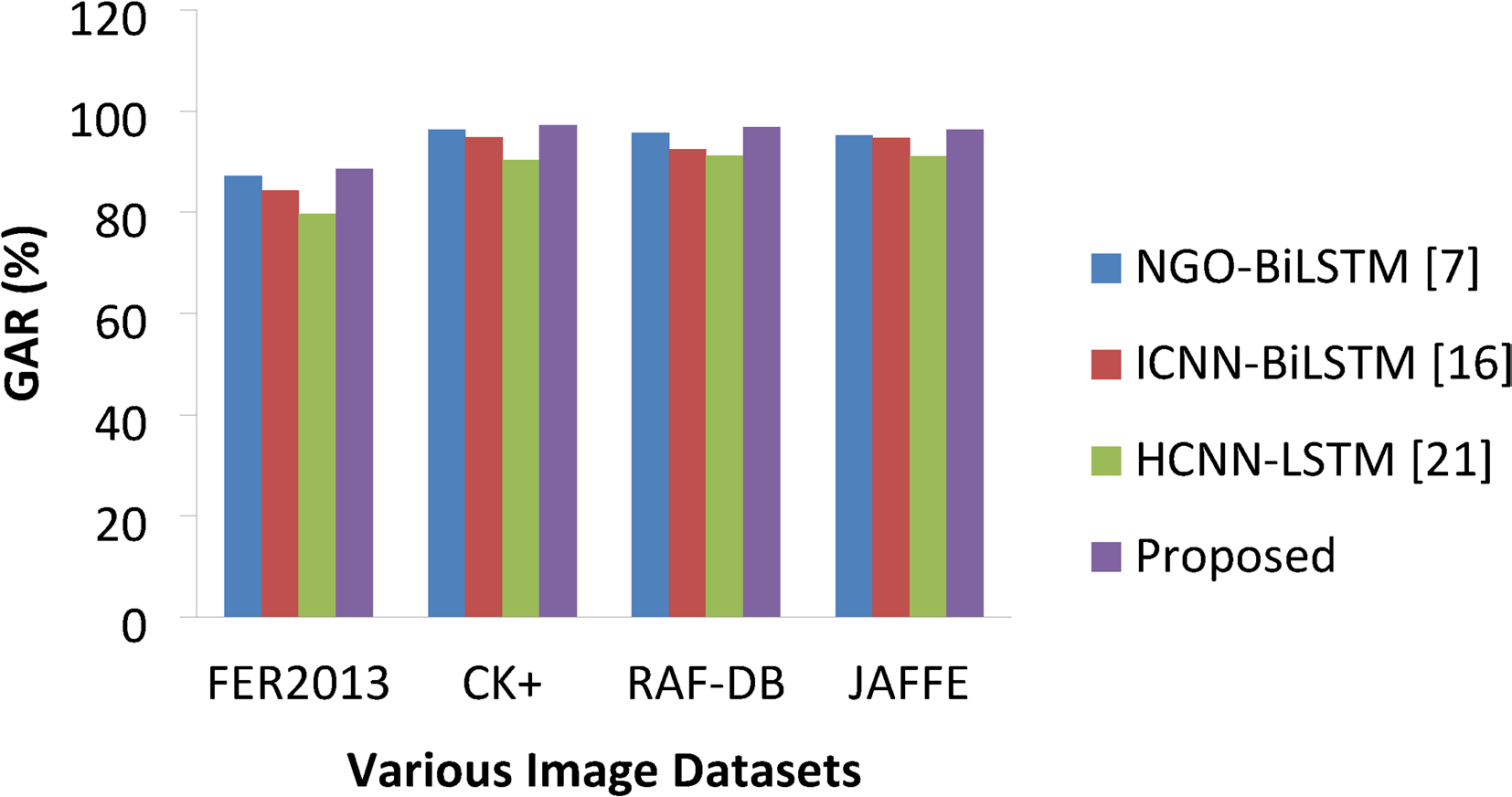
GAR percentages of various approaches.

**Fig. 15:**
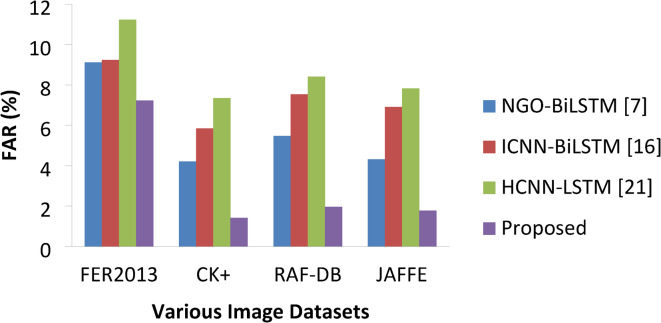
FAR percentages of various approaches.

Figures 14 and 15 represents the GAR and FRR of different models of this study on each datasets and the same if given in Tables 9 and 10. These metrics used to measure the success recognition rate and false recognition rate of each model on all three datasets. In general, the value of GAR must be as high as possible and the value of FRR must be as low as possible. The proposed model has achieved high GAR than other methods on each image class expressions with 88.57%, 97.23%, 96.87 and 96.32% on FER2013, CK+, RAF-DB and JAFFE respectively. The next best was delivered by NGO-BiLSTM which is followed by ICNN-BiLSTM and HCNN-LSTM finds last place. In the same way, worst FRR was produced by HCNN-LSTM with 11.23%, 7.35%, 8.41% and 7.82% on each datasets whereas proposed model resulted in lower FRR values of 7.23% on FER 2013, 1.42% on CK+, 1.96% on RAF-DB and 1.78% on JAFFE datasets. Thus, the overall results showed that the proposed DAM based DCNN with Bi-LSTM has outperformed other benchmarking approaches like NGO-BiLSTM, ICNN- BiLSTM and HCNN-LSTM on all performance metrics considered in this research study.

Thus, with the proposed approach we have achieved superior performance over other considered state-of-the-art approaches in this study in terms of accuracy and accuracy loss factors and also with performance evaluation metrics, especially, Genuine Acceptance Rate and False Recognition Rate with which we were able to figure the exact positive recognition of expressed emotions and identified exact percentage of false recognition as well. These metrics would help us to enhance the proposed model to improve the metric values further, especially on FER2013 dataset images.

## Conclusion and further research

Facial Expression Recognition (FER) is one of the most researched areas in recent decades. Variety of algorithms and models of deep learning techniques were employed and proposed to improve the expression recognition rate accuracy and reduce false expression recognition rate. This paper has proposed a single and cross fusion attention mechanism based DCNN with Bi-LSTM using piecewise cubic polynomial and linear activation function to integrate both local and global features of reconstructed images. Then, Global average pooling was used to compute weights for these feature vector maps and softmax layer classifies the images into 7 classes based on the expression present on particular image. The proposed model was compared with other benchmarking images such as NGO-BiLSTM, ICNN-Bi-LSTM, HCNN-LSTM with seven performance metrics and the results have showed that the proposed model has significant improvement over other models of the study in terms of various performance evaluation metrics. In terms of accuracy, the proposed DCNN-Bi-LSTM model achieved accuracy 82.89%, 96.78% and 95.78% and 95.87% on FER2013, CK+, RAF-DB and JAFFE datasets respectively. In terms of GAR, our model has achieved 88.57%, 97.23%, 96.87% and 96.32%, and FAR percentage stands at as low as 7.23% for FER2013, 1.42% for CK+, 1.96% for RAF-DB and 1.78% for JAFFE. Our proposed model has achieved expected results than the other models as the hybrid combination of DCNN with Bi-LSTM was effectively modeled, trained and implemented with techniques, methods discussed in this study have enhanced the recognition accuracy results and overall performance, and also avoided over-fitting issues, reduced computational power cost and complexity.

For further research, we could effectively consider General Adversarial Networks with Binary Attention Mechanism, Adam Optimizer (AO) and k-10 fold validation and compare the results with the model proposed in this paper and other recent state of the approaches in FER. Since the generator and discriminator of GAN model can help to extract more relevant and enhanced features from the input dataset images. Using a residual network with generator of GAN would help to reduce the amount of calculation and computation complexity. In addition, transformer based models like SWIN and vision transformer could also be considered since it can attend the entire image at once without considering the landmarks. Additionally, other datasets like AffectNet can be considered alongside of the datasets of this research study which would help to validate the generalizability of the proposed approach more accurately.

## Data Availability

CK+ dataset: https://www.kaggle.com/datasets/davilsena/ckdatasetRAF-DB dataset: https://www.kaggle.com/datasets/shuvoalok/raf-db-datasetJAFFE dataset: https://zenodo.org/records/14974867FER 2013 dataset: https://www.kaggle.com/c/challenges-in-representation-learning-facial-expression-recognition-challenge/dataThe data that support the findings of this study are available from the corresponding author, upon reasonable request.

## References

[1] Samar Elbedwehy, E. & Hassan, A. S. Rady elmonier, integrating neural networks with advanced optimization techniques for accurate kidney disease diagnosis. *Sci. Rep.* **14**, 21740. 10.1038/s41598-024-71410-6 (2024). 39289394 10.1038/s41598-024-71410-6 PMC11408592

[2] Esraa & Hassan Enhancing coffee bean classification: a comparative analysis of pre-trained deep learning models. *Neural Comput. Appl.* **36**, 9023–9052. 10.1007/s00521-024-09623-z (2024). (0123456789().,-volV).

[3] Esraa Hassan, A., Saber, S. & Elbedwehy Knowledge distillation model for acute lymphoblastic leukemia detection: exploring the impact of nesterov-accelerated adaptive moment Estimation optimizer. *Biomed. Signal Process. Control*. **94**, 106246. 10.1016/j.bspc.2024.106246 (2024).

[4] Jing Li, K., Jin, D., Zhou, N., Kubota, Z. & Ju Attention mechanism-based CNN for facial expression recognition, Neurocomputing, Volume 411, 21 October Pages 340–350, ISSN 0925–2312, (2020). 10.1016/j.neucom.2020.06.014

[5] Wenyun Sun, H., Zhao, Z. & Jin A visual attention based ROI detection method for facial expression recognition, Neurocomputing, Volume 296, 28 June 2018, Pages 12–22, ISSN 0925–2312. 10.1016/j.neucom.2018.03.034

[6] Ramachandran, B. & Rajagopal, S. D. 3D face expression recognition with ensemble deep learning exploring congruent features among expressions. *Comput. Intell.* **38**, 345–365. 10.1111/coin (2022).

[7] Zhong Jiarui, C. T. & Liuhan, Y. Face expression recognition based on NGO-BILSTM model. *Front. Neurorobotics.* **17**, 1662–5218. 10.3389/fnbot.2023.1155038 (2023). 10.3389/fnbot.2023.1155038 PMC10072256 37025255

[8] Li, H., Wang, N., Yu, Y., Yang, X. & Gao, X. LBAN-IL: A novel method of high discriminative representation for facial expression recognition, neurocomputing, **432**, Pages 159–169, ISSN 0925–2312, (2021). 10.1016/j.neucom.2020.12.076

[9] Li, S. & Deng, W. Reliable Crowdsourcing and Deep Locality-Preserving Learning for Unconstrained Facial Expression Recognition, in IEEE Transactions on Image Processing, vol. 28, no. 1, pp. 356–370, Jan. (2019). 10.1109/TIP.2018.2868382 10.1109/TIP.2018.2868382 30183631

[10] Melinte, D. O. & Vladareanu, L. Facial expressions recognition for Human–Robot interaction using deep convolutional neural networks with rectified Adam optimizer. *Sensors* **20**, 2393. 10.3390/s20082393 (2020). 32340140 10.3390/s20082393 PMC7219340

[11] Zhao, T. & Wu, X. Pyramid Feature Attention Network for Saliency Detection, 2019 IEEE/CVF Conference on Computer Vision and Pattern Recognition (CVPR), Long Beach, CA, USA, pp. 3080–3089, (2019). 10.1109/CVPR.2019.00320

[12] Li, Y., Zeng, J., Shan, S. & Chen, X. Occlusion aware facial expression recognition using CNN with attention mechanism. *IEEE Trans. Image Process.* **28**, 2439–2450. 10.1109/TIP.2018.2886767 (2018). 10.1109/TIP.2018.2886767 30571627

[13] Sherstinsky, A. Fundamentals of recurrent neural network (RNN) and long short-term memory (LSTM) network. *Phys. D: Nonlinear Phenom.* **404**, 132306 (2020).

[14] Guo, W., Wu, C., Ding, Z. & Zhou, Q. Prediction of surface roughness based on a hybrid feature selection method and long short-term memory network in grinding. *Int. J. Adv. Manuf. Technol.* **112**, 2853–2871. 10.1007/s00170-020-06523-z (2021).

[15] Jayaraman, S. & Mahendran, A. CNN-LSTM based emotion recognition using Chebyshev moment and K-fold validation with multi-library SVM. *PLoS ONE*. **20** (4), e0320058. 10.1371/journal.pone.0320058 (2025). 40193398 10.1371/journal.pone.0320058 PMC11975114

[16] Pansambal, B. H., Nandgaokar, D. A. B. & Rajput, D. J. L. Abhay wagh, an integrated CNN-BiLSTM approach for facial expressions. *Int. J. Adv. Comput. Sci. Appl. (IJACSA)*. **15** (3). 10.14569/IJACSA.2024.0150398 (2024).

[17] Mehendale, N. Facial emotion recognition using convolutional neural networks (FERC). *SN Appl. Sci.* **2**, 1–8. 10.1007/S42452-020-2234-1/TABLES/3 (2020).

[18] Han, J., Du, L., Ye, X., Zhang, L. & Feng, J. The devil is in the face: exploiting harmonious representations for facial expression recognition. *Neurocomputing* **486**, 104–113. 10.1016/j.neucom.2022.02.054 (2022).

[19] Gao, J., Li, L. & Guo, B. A. New extendface representation method for face recognition. *Neural Process. Lett.* **51**, 473–486. 10.1007/s11063-019-10100-1 (2020).

[20] Lihua & Lu Multi-angle face expression recognition based on generative adversarial networks. *Comput. Intell.* 1–18. 10.1111/coin.12437 (2021).

[21] Mohana, M., Subashini, P. & Krishnaveni, M. Emotion recognition from facial expression using hybrid CNN–LSTM network. *Int. J. Pattern Recognit. Artif. Intell.* **37** (8). 10.1142/S0218001423560086 (2023).

[22] Cao, T., Liu, C., Chen, J. & Gao, L. Nonfrontal and asymmetrical facial expression recognition through Half-Face frontalization and pyramid fourier frequency conversion. *IEEE Access. (Volume*. **9**, 17127–17138. 10.1109/ACCESS.2021.3052500 (2021).

[23] Dong, J., Zhang, Y. & Fan, L. A Multi-View face expression recognition method based on densenet and GAN. *Electronics* **12**, 2527. 10.3390/electronics12112527 (2023).

[24] Shen Hao, M. & Qinghao, L. Y. *Facial Expression Recognition by Merging Multilayer Features of Lightweight Convolutional Networks[J]*58610005 (Laser & Optoelectronics Progress, 2021). 6.

[25] Zhang, P. & Kong, W. Facial Expression Recognition Method Based on Multi-scale Efficient Channel Attention Mechanism, 2021 16th International Conference on Intelligent Systems and Knowledge Engineering (ISKE), Chengdu, China, 2021, pp. 680–686. 10.1109/ISKE54062.2021.9755441

[26] Lin, H., Ma, H., Gong, W., Wang, C. & Learning Non-frontal face recognition method with a side-face-correction generative adversarial networks, 2022 3rd International Conference on Computer Vision, Image and Deep & International Conference on Computer Engineering and Applications (CVIDL & ICCEA), Changchun, China, pp. 563–567, (2022). 10.1109/CVIDLICCEA56201.2022.9825237

[27] Radha Priyadharsini, G. & Krishnaveni, K. December, A novel framework using binary attention mechanism based deep Convolution neural network for face emotion recognition, measurement: sensors, 30, 100881, (2023). 10.1016/j.measen.2023.100881

[28] Liao, J. et al. Facial expression recognition methods in the wild based on fusion feature of attention mechanism and LBP. *Sensors* **23** (4204). 10.3390/s23094204 (2023). 10.3390/s23094204 PMC10180539 37177408

[29] Pratishtha Verma, V. & Aggrawal and Jyoti Maggu. FExR.A-DCNN: Facial Emotion Recognition with Attention mechanism using Deep Convolution Neural Network. In Proceedings of the 2022 Fourteenth International Conference on Contemporary Computing (IC3-2022). Association for Computing Machinery, New York, NY, USA, 196–203. (2022). 10.1145/3549206.3549243

[30] Gursesli, M. C. et al. Facial emotion recognition (FER) through custom lightweight CNN model: performance evaluation in public datasets, in IEEE access, **12**, pp. 45543–45559, (2024). 10.1109/ACCESS.2024.3380847

[31] Karani, R., Jani, J. & Desai, S. FER-BHARAT: a lightweight deep learning network for efficient unimodal facial emotion recognition in Indian context. *Discov Artif. Intell.* **4**, 35. 10.1007/s44163-024-00131-6 (2024).

[32] Debnath, T. et al. Four-layer ConvNet to facial emotion recognition with minimal epochs and the significance of data diversity. *Sci. Rep.* **12**, 6991. 10.1038/s41598-022-11173-0 (2022). 35484318 10.1038/s41598-022-11173-0 PMC9050748

[33] Rio Febrian, B. M., Halim, M., Christina, D., Ramdhan, A. & Chowanda, E. L. S. E. V. E. I. R. Volume 216, Pages 39–47, (2023). 10.1016/j.procs.2022.12.109

[34] Gao, J. et al. Dual-path facial emotion recognition based on attention mechanism, 2024 5th International Conference on Computer Vision, Image and Deep Learning (CVIDL), Zhuhai, China, 2024, pp. 554–558, 19–21 April 2024. 10.1109/CVIDL62147.2024.10603717

[35] Zhang, S., Zhang, Y., Zhang, Y., Wang, Y. & Song, Z. A. Dual-Direction attention mixed feature network for facial expression recognition. *Electronics* **12**, 3595. 10.3390/electronics12173595 (2023).

[36] Qian, Z. et al. Facial expression recognition based on strong attention mechanism and residual network. *Multimedia Tools Appl.* **82**, 14287–14306. 10.1007/s11042-022-13799-8 (2023).

[37] Zhang, F., Chen, G., Wang, H. & Zhang, C. Facial-expression recognition based on cross-fusion dual-attention network. *Comput. Visual Media*. **10** (3), 593–608. 10.1007/s41095-023-0369-x (June 2024).

[38] Lucey, P. et al. The extended cohn-kanade dataset (ck+): A complete dataset for action unit and emotion-specified expression, in IEEE computer society conference on computer vision and pattern recognition-workshops. IEEE, 2010, pp. 94–101. (2010).

[39] Nguyen, H. D. et al. Facial emotion recognition using an ensemble of multilevel convolutional neural networks. *Int. J. Pattern Recognit. Artif. Intell.* **33** (11), 1940015 (2019).

[40] Li, S., Deng, W., Du, J. & Recognition, P. IEEE Conference on Computer Vision and Reliable Crowdsourcing and Deep Locality-Preserving Learning for Expression Recognition in the Wild, (CVPR), Honolulu, HI, USA, 2017, pp. 2584–2593, (2017). 10.1109/CVPR.2017.277

[41] Lyons, M.J., Kamachi, M. & Gyoba, J. Coding facial expressions with Gabor wavelets (IVC special issue). 10.48550/arXiv.2009.05938 (2020).

[42] Lyons, M.J. “Excavating AI” re-excavated: debunking a fallacious account of the JAFFE dataset. arXiv 2107.13998. 10.48550/arXiv.2107.13998 (2021).

[43] Jain, N., Kumar, S. & Kumar, A. Effective approach for facial expression recognition using hybrid square-based diagonal pattern geometric model. *Multimed Tools Appl.* **78**, 29555–29571. 10.1007/s11042-019-7325-x (2019).

