# Correction: An interactive information based DCNN-BiLSTM model with dual attention mechanism for facial expression recognition

**DOI:** 10.1038/s41598-026-57390-9

**Published:** 2026-06-16

**Authors:** Samanthisvaran Jayaraman, Anand Mahendran

**Affiliations:** 1 School of Computer Science and Engineering, Vellore Institute of Technology, Vellore, Tamilnadu India; 2 School of Computer Science and Engineering, Vellore Institute of Technology, Chennai Campus, Vellore, Tamilnadu India

Correction to: *Scientific Reports* 10.1038/s41598-025-09709-1, published online 19 July 2025

The original version of this Article contained an error in the Proposed methodology section, under the subheading ‘Datasets’, where two references were omitted. The references are listed below as Reference 41 and 42.

41. Lyons, M.J., Kamachi, M. & Gyoba, J. Coding facial expressions with Gabor wavelets (IVC special issue). *arXiv* **2009.05938** 10.48550/arXiv.2009.05938 (2020).

42. Lyons, M.J*.* “Excavating AI” re-excavated: debunking a fallacious account of the JAFFE dataset. *arXiv* **2107.13998** 10.48550/arXiv.2107.13998 (2021).

As a result,

“Japanese Female Facial Expression (JAFFE) dataset have 213 images captured from 10 individual females with different universal facial expressions.”

now reads:

“Japanese Female Facial Expression (JAFFE) dataset^41,42^ have 213 images captured from 10 individual females with different universal facial expressions.”

Consequently, all subsequent References have been renumbered.

The original Article has been corrected.

